# Findings on three endocommensal scuticociliates (Protista, Ciliophora) from freshwater mollusks, including their morphology and molecular phylogeny with descriptions of two new species

**DOI:** 10.1007/s42995-024-00230-4

**Published:** 2024-05-21

**Authors:** Tao Li, Tengyue Zhang, Mingjian Liu, Zhe Zhang, Jiachen Zhang, Junhua Niu, Xiangrui Chen, Saleh A. Al-Farraj, Weibo Song

**Affiliations:** 1 Institute of Evolution and Marine Biodiversity, Ocean University of China and Key Laboratory of Evolution and Marine Biodiversity (Ministry of Education), Qingdao, 266003 China; 2 The Key Laboratory of Zoological Systematics and Application, College of Life Sciences, Hebei University, Baoding, 071002 China; 3 College of Marine Life Sciences, Ocean University of China, Qingdao, 266003 China; 4 School of Marine Sciences, Ningbo University, Ningbo, 315800 China; 5 Zoology Department, College of Science, King Saud University, 11451 Riyadh, Saudi Arabia; 6 Laboratory for Marine Biology and Biotechnology, Laoshan Laboratory, Qingdao, 266237 China

**Keywords:** Ciliates, Endocommensal, ITS2 secondary structure, Mollusks, New taxa, Wetland

## Abstract

Species of the ciliate genera *Myxophyllum* and *Conchophthirus* are found as endocommensals of terrestrial and freshwater mollusks, respectively. So far, there have been few studies of these genera and morphological data for most members are often incomplete. In the present work, two new species, *Myxophyllum weishanense* sp. nov. and *Conchophthirus paracurtus* sp. nov., and a known species, *Conchophthirus lamellidens*, were isolated from hosts in Lake Weishan Wetland, China. Taxonomic studies indicate that *M. weishanense* sp. nov. can be recognized mainly by the combination of about 60 somatic kineties on both ventral and dorsal sides and the presence of caudal cilia. *Conchophthirus paracurtus* sp. nov. differs from congeners in its body shape and size, having a glabrous area on the posterior right side, and having fewer somatic kineties. In addition, differences in their ITS2 (Internally Transcribed Spacer 2) secondary structures support the discrimination of the two new species from their highly similar congeners. An improved diagnosis for the poorly known species, *C. lamellidens* is also provided. Phylogenetic analyses reveal that members of the genus *Myxophyllum* belong to a fully supported clade that is sister to a large, poorly supported clade consisting of Hemispeiridae, Ancistridae, and several lineages of the nonmonophyletic Cyclidiidae. The *Myxophyllum* clade also includes *Protophyra ovicola* JQ956552, a possible misidentification. Sequences of the two new *Conchophthirus* species cluster with other congeners in a fully supported clade that is unrelated to either the ‘typical’ thigmotrichs or to pleuronematids, thus conflicting with the traditional classification, and may represent an orphan scuticociliate lineage.

**Supplementary Information:**

The online version contains supplementary material available at 10.1007/s42995-024-00230-4.

## Introduction

The phylum Ciliophora Doflein, 1901 (ciliates) is a highly diverse assemblage of single-celled eukaryotic microbes. Ciliates are heterotrophic or mixotrophic and exhibit complex cortex, nuclear dimorphism (macro- and micronuclei) and sexual reproduction by conjugation as synapomorphies (Gao et al. 2016; Lynn 2008). They are remarkably diverse, comprising around 8000 morphospecies and displaying a widespread geographic distribution (Adl et al. 2019; Lynn 2008). While the majority of ciliates are free-living in aquatic and terrestrial habitats (de Puytorac et al. 1994; Hu et al. 2019; Song et al. 2009; Wang et al. 2022), a substantial portion, encompassing at least 2600 species, are symbionts (Corliss 2000). Symbiotic ciliates are present in a broad spectrum of invertebrate and vertebrate hosts, including mollusks, which are one of the most broadly distributed and diverse invertebrate groups (Irwin and Lynn 2015; Lu et al. 2023; Lynn et al. 2018; Mayén-Estrada et al. 2021; Prosser et al. 2018; Raabe 1967, 1970, 1971, 1972; Song et al. 2003; Souidenne et al. 2019; Van As and Basson 2004; Zhang and Vďačný 2021, 2022, 2023a, b, 2024).

Two endocommensal scuticociliate genera, *Myxophyllum* (Stein, 1861) Raabe, 1934 and *Conchophthirus* Stein, 1861, have been known for over a century, but detailed taxonomic features such as the oral and somatic ciliature, remain unclear since most taxa have not been studied by modern methods. To date, all *Myxophyllum* populations have been identified as the same species, namely *M. steenstrupi* (Stein, 1861) Raabe, 1934, although they were collected from different hosts and differ from each other in some morphological features (de Puytorac et al. 1992; Kazubski 1964, 1973; Penn 1958; Raabe 1971; Stein 1861). Zhang and Vďačný (2021) carried out a complete redescription of the Slovak population of *M. steenstrupi* and provided the first sequences of the nuclear ribosomal RNA gene and mitochondrial cytochrome *c* oxidase I gene of this genus. This allowed us to investigate whether the *Myxophyllum* populations represent different species, i.e., whether there are cryptic species in *M. steenstrupi*.

The genus *Conchophthirus* Stein, 1861 mostly inhabits the mantle cavity of freshwater unionid mollusks (Antipa et al. 2020; Engelmann 1862; Ghosh 1918; Kahl 1931; Raabe 1933, 1934; Uyemura 1935; Zhang and Vďačný 2024). According to Raabe (1971), the genus *Conchophthirus* contains ten valid morphospecies, seven of which have illustrations based on silver nitrate-stained specimens, while all *Conchophthirus* species lack information on the oral ciliature. Antipa and Small (1971a) revealed the oral ciliature of *C. curtus* for the first time. More recently, Zhang and Vďačný (2024) documented the oral structure of *C. unionis* and *C. acuminatus*. To date, details of the oral ciliature, a crucial taxonomic feature, are lacking in the other seven species.

The role played by endocommensal ciliates in mollusks remains unclear, and collecting them is difficult as compared to free-living species. Consequently, few studies have focused on the diversity of endocommensal ciliates in recent decades, resulting in outdated and limited research data on them. In the present work, three mollusk-dwelling scuticociliates, one *Myxophyllum* species and two *Conchophthirus* species collected from the Lake Weishan Wetland in northern China, were thoroughly characterized using a morpho-molecular approach. Based on both morphological features and molecular data, two new species were established, namely *Myxophyllum weishanense* sp. nov. and *Conchophthirus paracurtus* sp. nov., while an improved redescription of *Conchophthirus lamellidens* was provided. Our multifaceted strategy also allows us to better understand the diversity and the evolutionary trajectory of *Conchophthirus* revealing that this genus is not related either to ‘traditional’ thigmotrichs or to pleuronematids, but rather represents an orphan lineage. In contrast, *Myxophyllum* is nested deep within the order Pleuronematida along with other ‘traditional’ thigmotrichs and some non-thigmotrich taxa.

## Materials and methods

### Collection and isolation

**Fig. 1:**
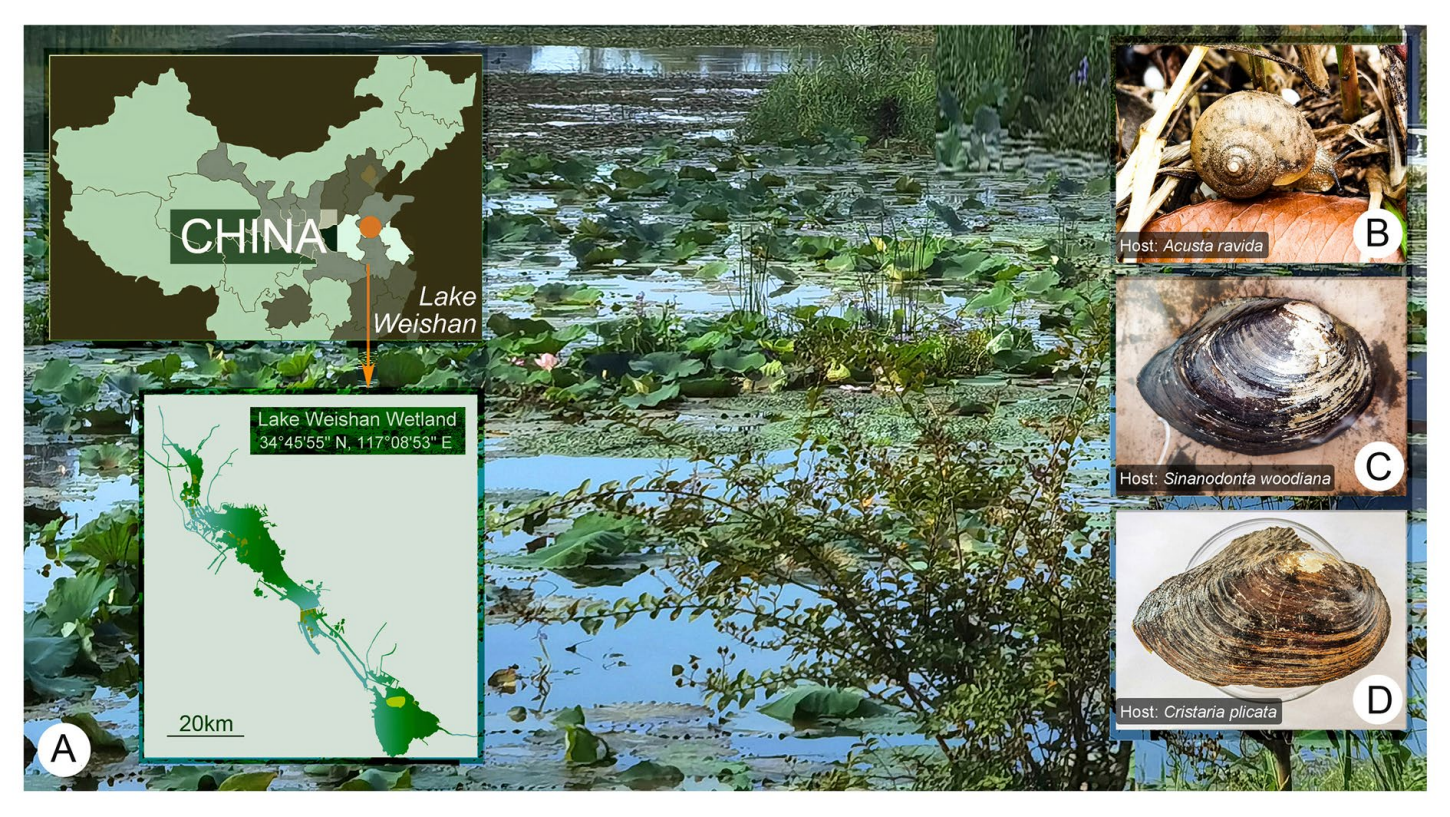
Sampling site and host mollusks. **A** The landscape of the sampling site, and partial map of China. Red dot marks the location of the Lake Weishan Wetland. **B** Snail *Acusta ravida*, host of *Myxophyllum weishanense* sp. nov. **C** Chinese pond mussel *Sinanodonta woodiana*, the host of *Conchophthirus lamellidens*. **D** Cockscomb pearl mussel *Cristaria plicata*, the host of *Conchophthirus paracurtus* sp. nov.

The host freshwater mussels and terrestrial snails were collected from the Lake Weishan Wetland, northern China (34°45′55′′ N, 117°08′53′′ E) (Fig. 1A). They were identified according to He and Zhuang (2013) and Chen and Zhang (2004), respectively.

*Myxophyllum weishanense* sp. nov. was found in the mantle cavity of the snail *Acusta ravida* Benson, 1842. Fifteen snails were collected from the humus on the river bank on June 18, 2021 (Fig. 1B). The humus temperature was 24 °C. Samples were observed in 14 of the 15 hosts and each contained about 20–100 ciliates. Twenty-four hours after detaching from their host, about half of the ciliate cells died, the remaining individuals exhibited reduced motility and their bodies became rounded, and all died after about three days.

Three individuals of the freshwater Chinese pond mussel *Sinanodonta woodiana* (Lea, 1834) were collected from a shallow river channel on March 25, 2021 (Fig. 1C), when the water temperature was 16 °C. *Conchophthirus lamellidens* was found in extremely high numbers (> 500 cells per host) in the mantle cavity of one of the three individuals, but fewer (about 50 cells per host) in two other individuals. Most *C. lamellidens* specimens were attached to the mantle cavity and gills.

Six individuals of the cockscomb pearl mussel, *Cristaria plicata* (Leach, 1815), were collected from a fish-culturing pond on November 24, 2021 (Fig. 1D), the water temperature was 9 °C. *Conchophthirus paracurtus* sp. nov. was found in moderate abundance (about 150 cells per host) within the mantle cavity and gills of all six mussel individuals.

### Morphological studies

Living ciliates were observed and photographed using bright field and differential interference contrast microscopy at 100–1000 × magnification. The ciliary pattern and the nuclear apparatus were revealed with the protargol staining method (Wilbert 1975). The silver nitrate staining method (Corliss 1953) was used to reveal the silverline system. Counts and measurements were made under 400–1000 × magnifications. Illustrations of living cells were produced from freehand sketches based on the photographs, while those of protargol- and silver nitrate-stained specimens were made using Adobe Photoshop according to the photomicrographs. Terminology is mainly according to Lynn (2008).

### DNA extraction, PCR amplification and sequencing

Three cells of each species were isolated for DNA extraction. All cells were washed five times with filtered in situ water (0.22 µm, Millex-GP filter unit) to exclude contamination (Liu et al. 2022). Genomic DNA was extracted using the DNeasy Blood & Tissue kit (Qiagen, Germany) following the optimized manufacturer’s protocol, with 25% of the suggested volume used for each solution. The primers 82-F (5′-GAA ACT GCG AAT GGC TC-3′) and ITS-R (5′-TAC TGA TAT GCT TAA GTT CAG CGG-3′) were used for PCR amplifications of the 18S rRNA gene and ITS1-5.8S-ITS2 region (Gao et al. 2012; Jerome et al. 1996). To minimize the possibility of PCR amplification errors, Q5^®^ Hot Start High-Fidelity 2 × Master Mix DNA Polymerase (New England BioLabs, USA) was used (Li et al. 2023). The thermal cycler program used was that described by Li et al. (2022). The quality of the amplified DNA was checked by 1% agarose gel electrophoresis. PCR products were purified using the EasyPure R Quick Gel Extraction Kit (TransGen Biotech Co., Ltd., Beijing, China), and then sequenced on an ABI-PRISM 3730 automatic sequencer (Applied Biosystems, Tsingke Biological Technology Company, Qingdao, China).

### Phylogenetic analyses

The 18S rRNA gene sequences of *Myxophyllum weishanense* sp. nov., *Conchophthirus lamellidens*, and *Conchophthirus paracurtus* sp. nov. were aligned with 132 other sequences downloaded from GenBank for phylogenetic analyses (for all taxa and accession numbers, see Supplementary Table S1). Four colpodeans, i.e., *Colpoda lucida*, *Colpoda magna*, *Maryna umbrellata*, and *Platyophrya bromelicola,* were selected as the outgroup. All sequences were aligned with the MAFFT ver. 7 server (https://mafft.cbrc.jp/alignment/server/) (Katoh et al. 2019), using the iterative refinement E-INS-i method, the 200PAM/k = 2 scoring matrix, and the gap opening penalty at 1.53. Primer sequences were removed but otherwise the 18S rRNA gene sequence alignment was not masked. The final alignment, comprising 1762 positions, was used for the phylogenetic analyses. The number of unmatched nucleotides, and the identity of the three newly submitted sequences with their most closely sequences, were calculated according to Li et al. (2021). The number of unmatched nucleotides and the pairwise *p*-distances of five *Conchophthirus* species were calculated with the program BioEdit ver. 7.0 (Hall 1999), using the sequence difference count matrix and sequence identity matrix options.

Maximum likelihood (ML) analysis was performed with 1000 bootstrap replicates to estimate the reliability of the internal branches using the program IQTREE ver. 1.6.10 (Nguyen et al. 2015) on the IQ-TREE web server (https://iqtree.cibiv.univie.ac.at/) (Trifinopoulos et al. 2016). Bayesian inference (BI) was performed using MrBayes v3.2.7 (Ronquist et al. 2012) with the best-fit model GTR + I + G, selected using the IQ-TREE web server. Altogether 5,000,000 generations with a sampling frequency of 100 and a relative burn-in fraction of 25% of sampled trees were generated. All the remaining trees were used to calculate posterior probabilities (PP) using a 50% majority rule consensus. The other parameters were used at default settings. Convergence of the Markov Chain Monte Carlo (MCMC) analyses was assessed as follows: after 5,000,000 generations, the average standard deviation of split frequencies was < 0.01, the potential scale reduction factor approached 1, effective sample sizes were greater than 200, and no obvious trends were recognizable in the plots of generations versus log probability. All trees were computed as unrooted and were rooted in FigTree v.1.2.3 (https://tree.bio.ed.ac.uk/software/figtree/), using out-group taxa as specified above.

The interpretation of the bootstrap values follows Vďačný and Rajter (2015), that is, values < 70% were considered as low, from 70 to 94% as moderate, and ≥ 95% as high (Hillis and Bull 1993). Following Alfaro et al. (2003), Bayesian posterior probability < 0.95 was considered as low and ≥ 0.95 as high.

### Prediction of ITS2 secondary and tertiary structure

The boundaries of the ITS2 region were determined by constructing the secondary structure of ITS-5.8S rRNA region using R2DT (RNA 2D Templates) web server (https://rnacentral.org/r2dt) (Sweeney et al. 2021) and searching for the highly characteristic 5.8S-28S rRNA proximal stem. Formation of the hybridized 5.8S-28S rRNA proximal stem was forced and all other parameters were left at default settings. Homologies of the secondary structures of the predicted thermodynamically optimal ITS2 region were compared with consensus ITS2 secondary structures of the most closely related oligohymenophorean ciliates (Miao et al. 2008; Zhang and Vďačný 2021). The putative models were then prepared in VARNA v.3.93 (Darty et al. 2009). The consensus structure of the ITS2 region for five *Conchophthirus* species was also calculated in 4SALE (Seibel et al. 2006).

## Results

### ZooBank registration

- Present work: urn:lsid:zoobank.org:pub:A13507D1-5038-4315-8184-27AB1943FDBC
- *Myxophyllum weishanense* sp. nov.:
- urn:lsid:zoobank.org:act:54B40B3D-61D2-4E91-963F-F21919740867
- *Conchophthirus lamellidens* Ghosh, 1918:
- urn:lsid:zoobank.org:act:53CEBD5D-93C8-4D6D-A4AB-19EA250969BF
- *Conchophthirus paracurtus* sp. nov.:
- urn:lsid:zoobank.org:act:892AA461-36B0-4AE3-A462-688B1AD91903

### Taxonomy

- Class Oligohymenophorea de Puytorac et al., 1974
- Subclass Scuticociliatia Small, 1967
- Order Pleuronematida Fauré-Fremiet in Corliss, 1956
- Family Thigmophryidae Chatton & Lwoff, 1926
- Genus *Myxophyllum* Raabe, 1934

### ***Myxophyllum weishanense*** sp. nov. (Figs. 2, 3; Table 1)

**Fig. 2:**
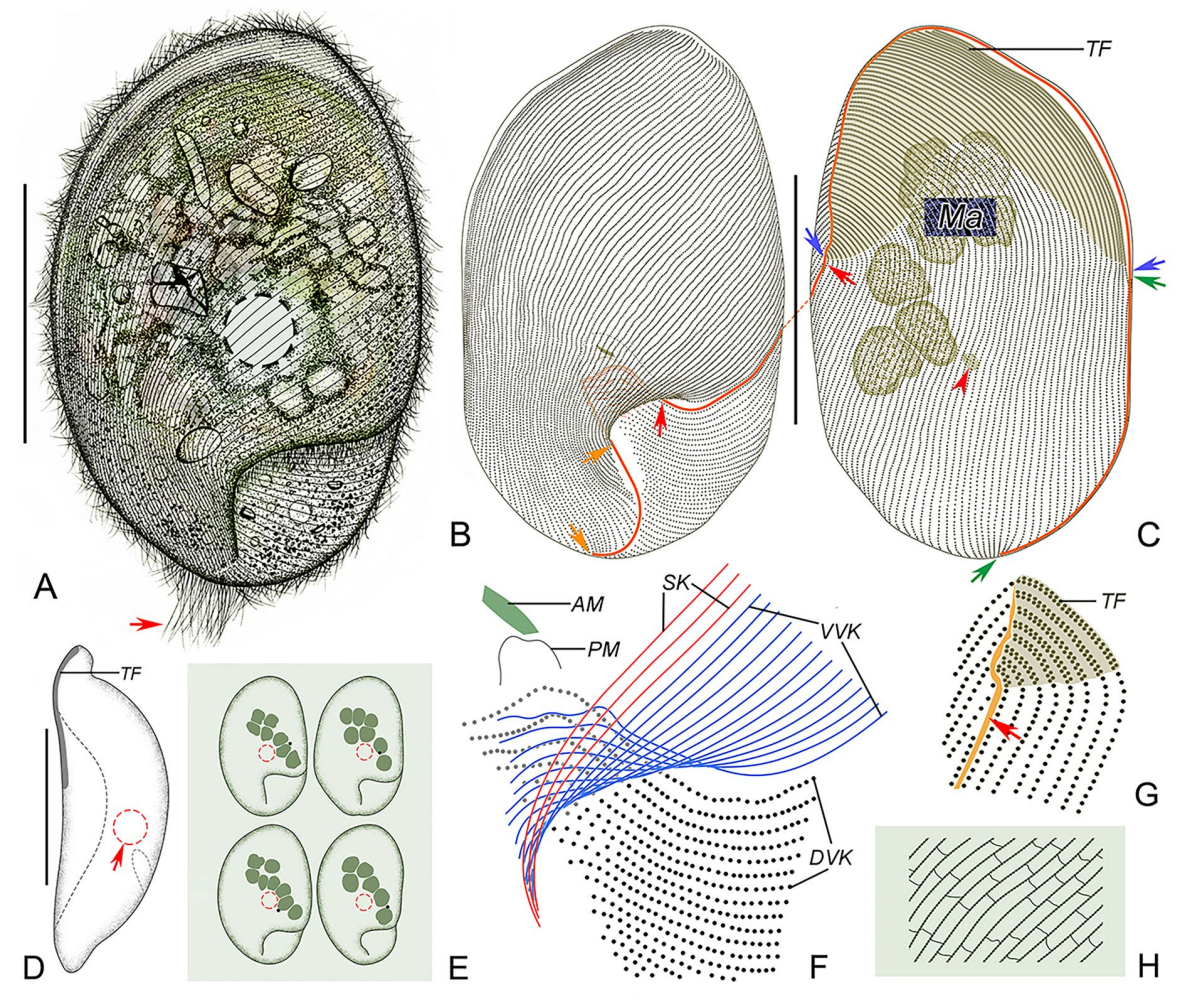
*Myxophyllum weishanense* sp. nov. in vivo (**A**, **D**, **E**), after protargol (**B**, **C**, **F**, **G**) and silver nitrate (**H**) staining. **A** Ventral view of a representative cell, arrow marks the caudal cilia. **B**, **C** Ventral (**B**) and dorsal (**C**) views, to show the ciliature and location of the nuclear apparatus. Blue arrows denote the anterior suture, green arrows mark the right lateral suture, red arrows show the left lateral suture, orange arrows mark the postoral (posterior) suture, red arrowhead denotes the micronucleus. **D** Lateral view, arrow shows the contractile vacuole. **E** Individuals with different body shapes. **F** Oral apparatus, the blue lines represent the somatic kineties wrapped within the mouth pocket, and the red lines represent the exposed somatic kineties. **G** SK on the left side of the dorsal body, red arrow marks the suture on the left side of the thigmotactic field. **H** Reticulate silverline system. *AM* adoral membranelle, *DVK* dorsal vestibular kineties, *Ma* macronuclei, *PM* paroral membrane, *SK* somatic kineties, *TF* thigmotactic field, *VVK* ventral vestibular kineties. Scale bars = 60 μm

**Fig. 3:**
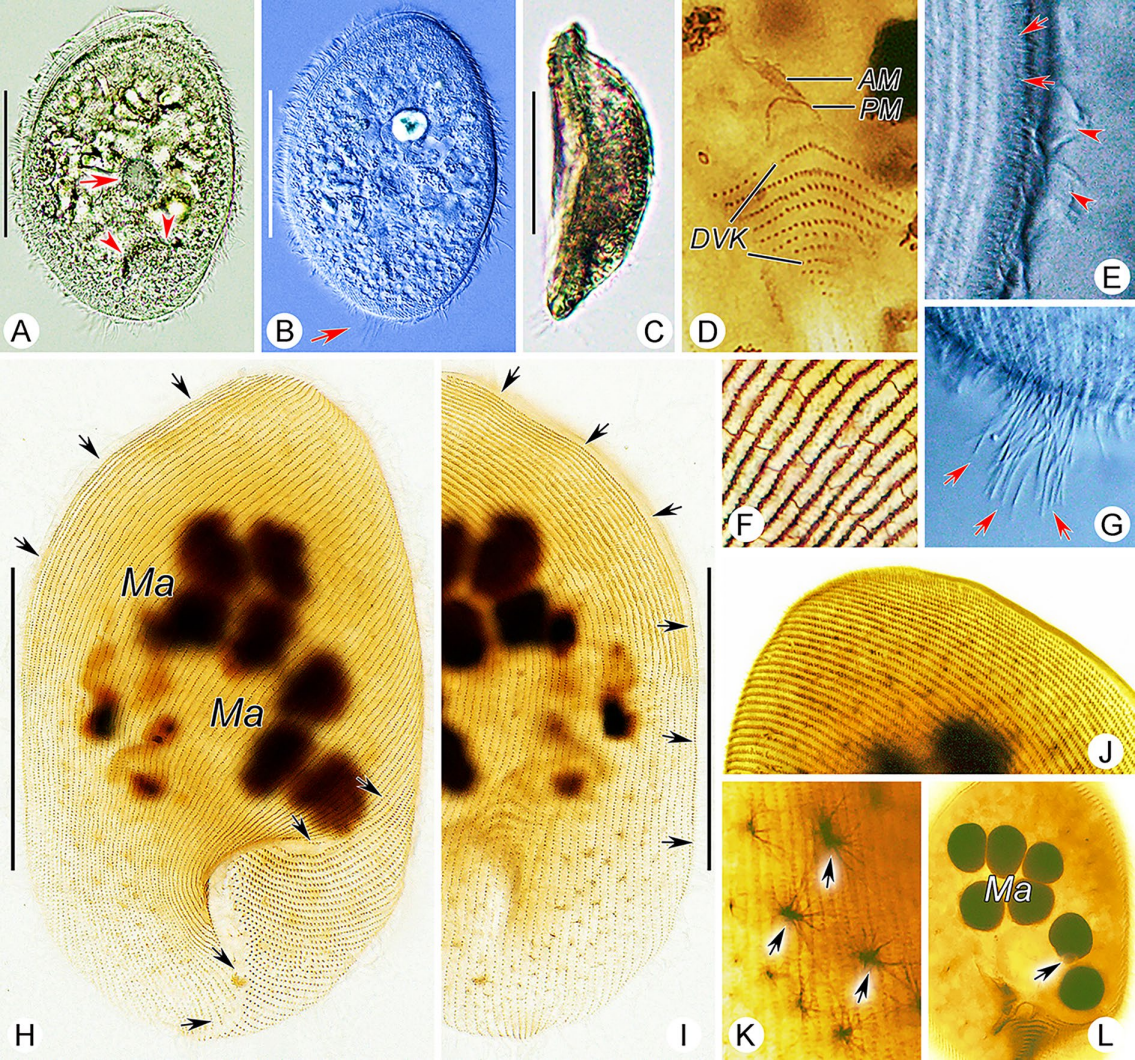
*Myxophyllum weishanense* sp. nov. in vivo (**A**–**C**, **E**, **G**), after protargol (**D**, **H**–**L**) and silver nitrate (**F**) staining. **A**, **B** Ventral view of two representative individuals, arrow in **A** shows the contractile vacuole, arrowheads show the opening of the mouth pocket; arrow in **B** shows caudal cilia. **C** Left-lateral view. **D** Oral apparatus. **E** Ventral–dorsal border of the anterior body region, arrows show the thigmotactic cilia, arrowheads show the somatic cilia. **F** Reticulate silverline system. **G** Arrows show the caudal cilia. **H**, **I** Ventral (**H**) and dorsal (**I**) views of the holotype specimen to show the ciliature, arrows show the anterior, right lateral, left lateral, and postoral sutures. **J** Thigmotactic field at anterior end of dorsal side. **K** Arrows show the fibrous structures beneath cortex. **L** Nuclear apparatus, arrow marks the micronucleus. *AM* adoral membranelle, *DVK* dorsal vestibular kineties, *Ma* macronuclei, *PM* paroral membrane. Scale bars = 60 μm

**Table 1:** Morphometrical characterization of *Myxophyllum weishanense* sp. nov. (Mwe, the 1st lines), *Conchophthirus lamellidens* (Cla, the 2nd lines) and *Conchophthirus paracurtus* sp. nov. (Cpa, the 3rd lines)

Character	Species	Min	Max	Mean	Median	SD	SE	CV	*n*
Body length (μm)	Mwe	86	153	114.5	116	18.7	4.2	16.3	20
	Cla	55	70	64.9	65	4.5	1	6.9	20
	Cpa	65	91	75.7	74.5	9.2	2.2	12.1	18
Body width (μm)	Mwe	53	100	74.8	76	13.1	2.9	17.6	20
	Cla	30	45	37.6	40	4.2	0.9	11.1	20
	Cpa	32	55	44.4	44.5	7.3	1.7	16.4	18
Number of macronuclei (μm)	Mwe	6	8	7	7	0.4	0.1	5.7	20
	Cla	1	1	1	1	0	0	0	20
	Cpa	1	1	1	1	0	0	0	18
Diameter of macronucleus (μm)	Cla	15	26	20.4	21	2.8	0.6	13.7	20
	Cpa	9	21	15.7	15.3	3.4	0.8	21.9	18
Number of somatic kineties (SK)	Mwe	111	122	118.6	119.5	2.7	0.6	2.2	20
	Cla	62	74	67.6	68	2.7	0.6	4	20
	Cpa	96	124	108.6	107	9.7	2.3	9	18
Number of SK on ventral side	Mwe	55	61	58.5	58.5	1.4	0.3	2.3	20
Number of SK on dorsal side	Mwe	56	62	60.2	60.5	1.6	0.4	2.7	20
Number of thigmotactic kineties	Mwe	56	62	60.2	60.5	1.6	0.4	2.7	20
	Cla	20	27	22.2	22	2.1	0.5	9.4	20
	Cpa	35	56	44.4	41.5	7.3	1.7	16.3	18
Number of ventral ventral kineties	Mwe	10	17	14.2	14	1.9	0.4	13.4	20
Number of dorsal ventral kineties	Mwe	6	10	7.4	7	1.4	0.3	18.8	20
Number of postoral kineties	Cla	1	1	1	1	0	0	0	20
	Cpa	2	2	2	2	0	0	0	12
Number of kineties in membranelle 1	Cla	3	3	3	3	0	0	0	20
	Cpa	2	2	2	2	0	0	0	12
Number of kineties in membranelle 2	Cla	3	3	3	3	0	0	0	20
	Cpa	2	2	2	2	0	0	0	12
Number of kineties in membranelle 3	Cla	3	3	3	3	0	0	0	20
	Cpa	2	2	2	2	0	0	0	12
Number of kineties in paroral membrane	Mwe	1	1	1	1	0	0	0	20
	Cla	1	1	1	1	0	0	0	20
	Cpa	1	1	1	1	0	0	0	12

*CV* coefficient of variation in %, *Max* maximum, *Mean* arithmetic mean, *Min* minimum, *n* number of specimens investigated, *SD* standard deviation, SE standard error

#### Diagnosis

Medium-sized *Myxophyllum*, size about 100–150 × 70–100 μm in vivo; oral apparatus reduced to a paroral membrane and one adoral membranelle, both sunken in buccal cavity; dorsal and ventral vestibular kineties not connected at mouth pocket; about 6–8 spherical macronuclei grouped together; 55–61 somatic kineties on ventral side composed of monokinetids, 56–62 somatic kineties on dorsal side, posterior parts of dorsal kineties composed of monokinetids, anterior parts composed of dikinetids, forming a prominent, arch-shaped, anterodorsal thigmotactic field; about 20–30 caudal cilia.

#### Etymology

The species-group name *weishanense* refers to the area (Weishan, China) where the sample was collected.

#### Type locality and habitat

Isolated from the mantle cavity of the snail, *Acusta ravida* (Benson, 1842), found in Lake Weishan Wetland (34°45′55′′ N, 117°08′53′′ E), northern China.

#### Deposition of type slide

The protargol-stained slide containing the holotype specimen marked with a red ink circle (Figs. 2B, C, 3H, I) and several paratype specimens marked with black ink circles (registration number: LT2021061801-1) is deposited in the Laboratory of Protozoology, Ocean University of China, Qingdao, China.

#### Gene sequence

The 18S rRNA gene sequence of *Myxophyllum weishanense* sp. nov. is deposited in GenBank with the accession number OR042378. The length and G + C content of the sequence are 1670 bp and 45.21%, respectively. The ITS1-5.8S rRNA-ITS2 region sequence is deposited in GenBank with accession number OR148434. The length and G + C content are 477 bp and 35.22%, respectively.

#### Description

Body size about 100–150 × 70–100 μm in vivo (Figs. 2A–C, 3A, B, H, I). Body oval to elliptical in outline, with anterior end inclined slightly to left of longitudinal axis (Figs. 2A, E, 3A, B). Length to width ratio about 1.5:1. Dorsoventrally flattened about 2:1, ventral side arched and dorsal side depressed in mid-region (Figs. 2D, 3C). Ventral side sunken in lower left region, occupying about 25% and 45% of body length and width, respectively and forming a buccal cavity (Figs. 2A, 3A). Buccal cavity positioned posteriorly, sunken into body and forming a mouth pocket (Figs. 2A, 3A). Cytoplasm colorless, contains densely distributed lipid droplets, about 3–5 μm in diameter (Fig. 3A). Large particles aggregated in body center, about 10–20 μm in length, possibly ingested host tissue (Figs. 2A, 3A, B). Protargol-stained specimens showed longitudinally oriented fibrous structures in cytoplasm, not discernible in vivo (Fig. 3K). Single contractile vacuole located near cell center, about 14–18 µm in diameter in diastole, contracts at about 10 s intervals, contractile vacuole pore not observed in vivo or in protargol preparations (Figs. 2A, D, 3A). Six to eight macronuclei grouped in center of anterior half of cell, oval, about 15 μm in length (Figs. 2C, E, 3H, L). Single micronucleus located adjacent to second macronucleus from posterior end, about 2–4 μm in diameter (Figs. 2C, 3L). No cortical granules or extrusomes observed.

Swimming motion of isolated cells slow, rotating about long axis, or by gliding slowly with dorsal side attached to substrate.

Somatic cilia about 6–8 μm long; arranged in narrowly-spaced somatic kineties; about 55–61 somatic kineties on ventral side and 56–62 on dorsal side (Figs. 2B, C, 3H, I). Posterior ends of ventral kineties terminate along postoral suture and right posterior cell margin, anterior parts of ventral kineties with left-hand spiral, terminating along left cell margin at suture anterior to level of mouth pocket (Figs. 2B, C, 3H, I). Posterior parts of dorsal kineties straight, terminating along posterior cell margin; anterior parts with left-hand spiral abutting with ventral kineties at anterior suture on left cell margin (Figs. 2B, C, 3H). Dorsal kineties similar to that on ventral side, anterior half of each kinety obliquely oriented, posterior half vertical; left half of dorsal somatic kineties conspicuously curved at posterior ends, extending onto ventral side and into buccal cavity, terminating along rightmost end of buccal cavity (Fig. 2C, G). All ventral kineties and posterior dorsal kineties consist of monokinetids. Anterodorsal dikinetids forming an irregular triangular thigmotactic field, recessed upwards at base, about two-fifths of body length, thigmotactic cilia about 3 µm long in vivo (Figs. 2B, C, G, 3E, H–J). Conspicuous suture running around body along ventral–dorsal border, commencing at left end of mouth pocket and terminating at right end of mouth pocket (Figs. 2B, C, G, 3H, I). Upper left suture broad, forming a nonciliated area (Figs. 2B, C, 3H, I). About 20–30 caudal cilia directed slightly to right, each about 15–20 µm long (Figs. 2A, 3B, G). Silverline system reticulate, silverlines extending to somatic kineties, with short, loose and irregular transverse silverlines connecting adjacent somatic kineties, forming closed rectangular grids (Figs. 2H, 3F).

Oral apparatus within mouth pocket on ventral side in posterior two-fifths of cell (Figs. 2B, F, 3D, 9E). Seven to ten leftmost dorsal kineties and 11–14 leftmost ventral kineties extend into mouth pocket and forming vestibular kineties; dorsal and ventral vestibular kineties not connected at mouth pocket (Figs. 2F, 3D). Oral apparatus reduced; paroral membrane short, inverted U-shape, single-rowed, situated anterior to dorsal vestibular kineties (Figs. 2F, 3D, 9E); adoral zone reduced to single rectangular adoral membranelle, situated anterior to paroral membrane (Figs. 2F, 3D, 9E).

- Class Oligohymenophorea de Puytorac et al., 1974
- Subclass Scuticociliatia Small, 1967
- Order Loxocephalida Jankowski, 1980
- Family Conchophthiridae Kahl in Doflein & Reichenow, 1929
- Genus *Conchophthirus* Stein, 1861

### *Conchophthirus lamellidens* Ghosh, 1918 (Figs. 4, 5; Table 1)

**Fig. 4:**
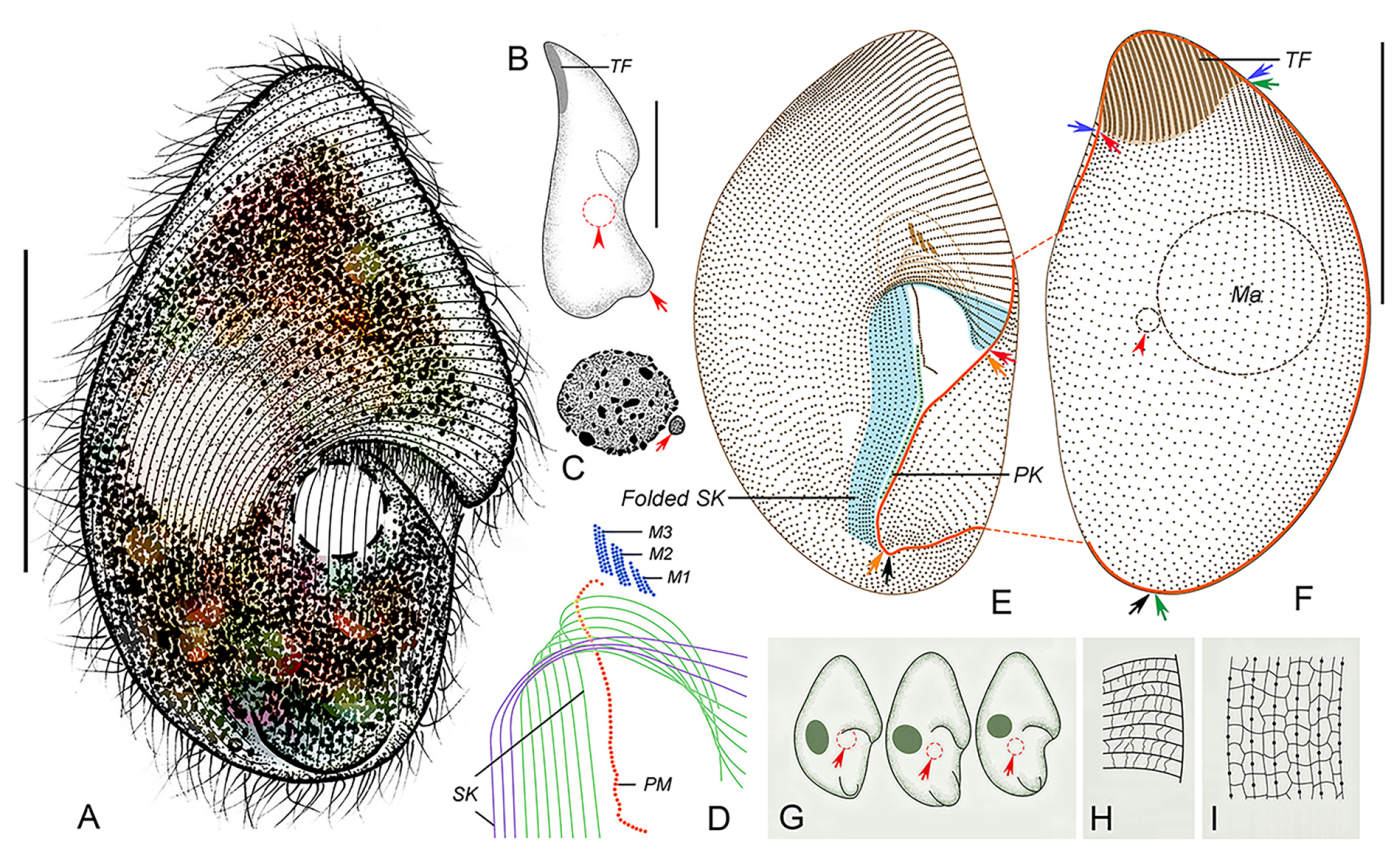
*Conchophthirus lamellidens* in vivo (**A**, **B**, **G**), after protargol (**C**–**F**) and silver nitrate (**H**, **I**) staining. **A** Right ventrolateral view of a representative cell. **B** Right-lateral view, arrow shows the bulge at posterior body end, arrowhead shows the contractile vacuole. **C** Nuclear apparatus, arrow shows the spherical micronucleus. **D** Oral apparatus, the green lines represent the SK wrapped within the mouth pocket, and the purple lines represent the exposed somatic kineties. **E**, **F** Ventral (**E**) and dorsal (**F**) views, to show the ciliature and location of the nuclear apparatus. Blue arrows denote the anterior suture, green arrows mark the right lateral suture, red arrows show the left lateral suture, orange arrows mark the postoral suture, black arrow mark a short transverse suture, red arrowhead denotes the micronucleus. **G** Variations of body shape, arrows show the contractile vacuole. **H**, **I** Silverline system for the anterior (**H**) and posterior (**I**) parts of the body. *M1–3* membranelle 1–3, *PK* postoral kinety, *PM* paroral membrane, *SK* somatic kineties, *TF* thigmotactic field. Scale bars = 45 μm (**A**, **E**), 30 μm (**B**)

**Fig. 5:**
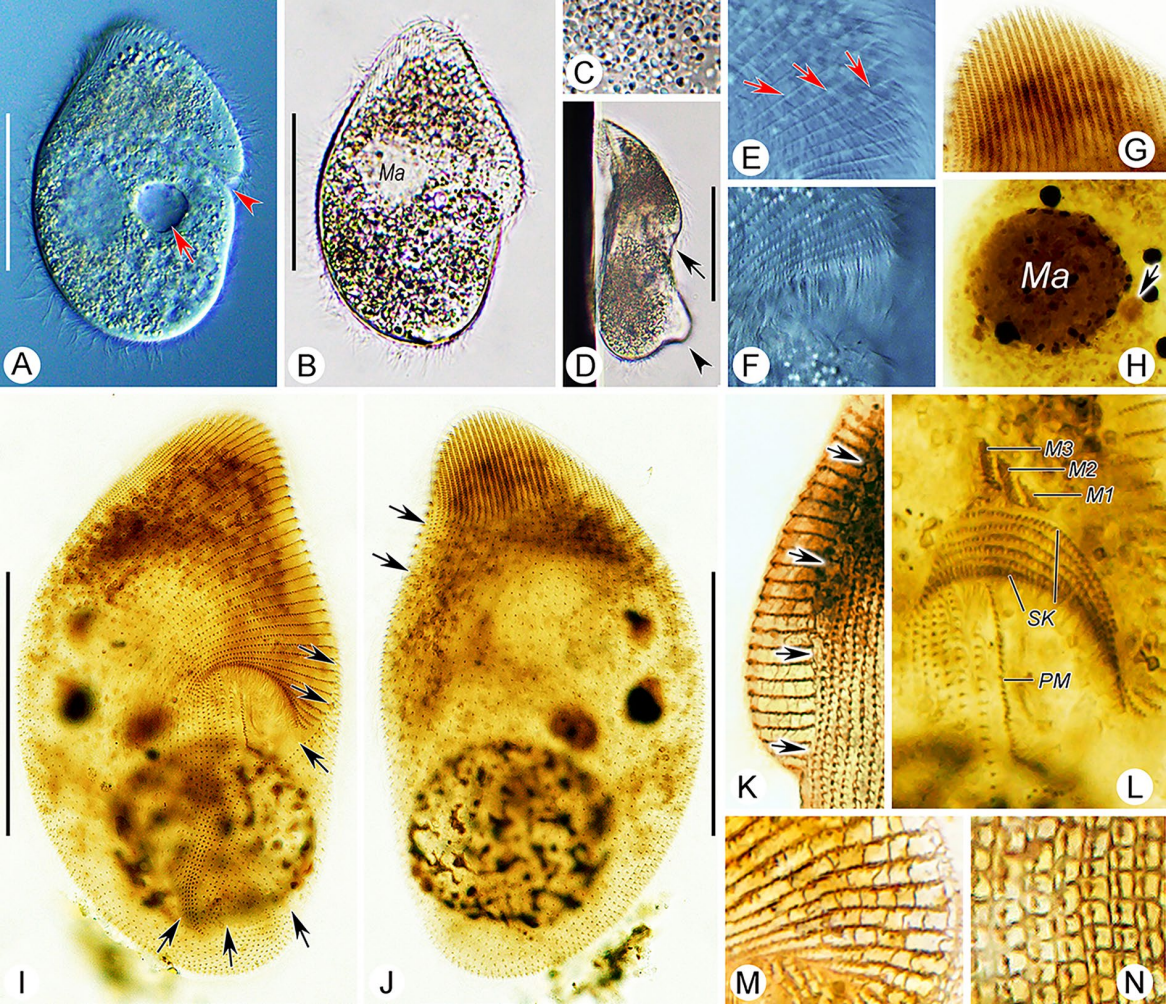
*Conchophthirus lamellidens* in vivo (**A**–**F**), after protargol (**G**–**J**, **L**) and silver nitrate (**K**, **M**, **N**) staining. **A** Right ventrolateral view of a slightly compressed cell, arrow shows the contractile vacuole, arrowhead shows the mouth pocket. **B** Right ventrolateral view of a representative cell. **C** Particles concentrated in the upper part of the body. **D** Lateral view of a representative cell, arrow shows the mouth pocket, arrowhead shows the bulge at posterior body end. **E** Cell surface, arrows show the ribs distributed along the somatic kineties. **F** Opening of the mouth pocket. **G** Thigmotactic field at the anterior region of dorsal side. **H** Nuclear apparatus, arrow shows the spherical micronucleus. **I**, **J** Ventral (**I**) and dorsal (**J**) views of the lectotype specimen to show the ciliature, arrows show the postoral and left lateral suture. **K** Arrows show the left lateral suture extending from the left of the mouth pocket to the dorsal side. **L** Oral apparatus. **M**, **N** Silverline system for the upper (**M**) and lower (**N**) parts of the body, respectively. *M1–3* membranelle 1–3, *Ma* macronucleus, *PM* paroral membrane, *SK* somatic kineties. Scale bars = 45 μm (**A**, **B**, **D**), 30 μm (**I**, **J**)

#### Remarks

This species was established by Ghosh (1918), and then Kahl (1931) redrew it based on Ghosh’s figure. Uyemura (1935) rediscovered it in Japan and briefly described the Japanese population. Raabe (1971) collated the historical description of *C. lamellidens* based on the available in vivo data. Hence, to date, this organism has never been studied in detail regarding its infraciliature and its taxonomic information remains incomplete. Here, we supply an improved diagnosis based on morphologic data from the Chinese population and previously reported populations. Due to the original description of *C. lamellidens* lacking information about holotype, and no neotype or lectotype designated in previous studies, all the specimens of the type series are automatically syntypes (ICZN 1999). According to the article 74.1 of ICZN (1999), we designate a Chinese specimen as the lectotype for *C. lamellidens*, and other specimens then become paralectotypes.

#### Improved diagnosis

Medium-sized *Conchophthirus*, about 80–110 × 35–65 μm in vivo; anterior end of cell conspicuously narrowed while posterior end is broadly rounded; single macronucleus located in body center with one or two adjacent micronuclei; single contractile vacuole positioned near left cell margin near equator; 62–74 somatic kineties, SK (somatic kinety) 1 to SK7–ninefold into mouth pocket; mouth pocket located near cell equator beneath a lobe-like protrusion of left cell margin; oral apparatus comprises a long, bracket-shaped paroral membrane and a transversely arranged group of three membranelles, each composed of three short rows; paroral membrane extending from caudal end of membranelle 3 to bottom of mouth pocket.

#### Type locality and habitat

Isolated from the mantle cavity and gills of *Sinanodonta woodiana* (Lea, 1834), found in Lake Weishan Wetland (34°45′55′′ N, 117°08′53′′ E), northern China.

#### Deposition of type slide

The protargol-stained slide containing the lectotype specimen marked with a red ink circle (Figs. 4E, F, 5I, J) and several paralectotype specimens marked with black ink circles (registration number: LT2021032501-1) is deposited in the Laboratory of Protozoology, Ocean University of China, Qingdao, China.

#### Gene sequence

The 18S rRNA gene sequence of *Conchophthirus lamellidens* is deposited in GenBank with accession number OR042379. The length and G + C content of the sequence are 1641 bp and 42.84%, respectively. The ITS1-5.8S rRNA-ITS2 region sequence (accession number OR148435) has a length and G + C content of 542 bp and 37.27%, respectively.

#### Description

Body size about 80–100 × 45–65 μm in vivo (Figs. 4A, 5A, B). Body variable in shape, with upper half triangular and lower half broad to narrow semi-elliptical in right ventrolateral view, caudal end with a prominent ventral bulge posteriorly (Figs. 4A, B, G, 5A, B, D). Length to width ratio about 1.5–2:1. Dorsoventrally compressed about 2:1. In lateral view, anterior end pointed and curved towards dorsal side, posterior end slightly broader, and mid-region of ventral surface concave (Figs. 4B, 5D). Oral apparatus located in deep vestibulum on left ventral cell margin, its opening located beneath a lobe-like protrusion in mid-region of left margin (Figs. 4A, E, 5A, B, F, I). No cortical granules or extrusomes observed. Cytoplasm colorless, grey particles about 2 μm in diameter usually concentrated in upper part of body giving cells grey-black color (Fig. 5B, C). Single contractile vacuole located to left of midline at cell equator, about 10–18 µm in diameter in diastole, contracts at about 50 s intervals (Figs. 4A, B, G, 5A). Single spherical macronucleus about 20 μm in diameter, located right of midline at cell equator; single micronucleus about 2–3 μm in diameter, adjacent to macronucleus (Figs. 4C, F, 5B, H).

Locomotion by gliding at moderate speed with dorsal side attached to substrate or by swimming at moderate speed while rotating about long body axis.

Somatic cilia about 8–10 μm long, arranged in 62–74 somatic kineties; ventral kineties with right-hand spiral in anterior region and more or less straight posteriorly; dorsal somatic kineties with slightlright-hand spiral; posterior ends of left 6–15 rows extend to ventral side to form a short transverse suture (Figs. 4E, F, 5I, J). Somatic kinety 1 to somatic kineties 7–ninefold into mouth pocket (Figs. 4D, E, 5I, L). All ventral kineties consist of monokinetids; dorsal kineties composed of monokinetids posteriorly and dikinetids anteriorly; triangular thigmotactic field on dorsal surface at anterior cell end, consisting of about 20–27 kineties, occupying about 20% of body length (Figs. 4E, F, 5I, J). Thigmotactic cilia about 6 µm long in vivo. Anterior suture begins on dorsal side of lobe-like projection, extends upward along ventral–dorsal margin to rightmost part of thigmotactic field (Figs. 4E, F, 5I, J, K). Postoral suture starts at base of mouth pocket, extends posteriorly to caudal bulge (Figs. 4E, 5I). Silverline system grid-like, formed by longitudinal primary meridians connecting basal bodies, longitudinal secondary meridians between kineties, and short transverse connectives between primary and secondary meridians (Figs. 4H, I, 5M, N).

Oral apparatus located inside mouth pocket (Figs. 4D, E, 5I, L, 9E). Three rhomboid adoral membranelles in a transversely arranged group; each membranelle composed of three rows; membranelles increase in length from membranelle 1 to membranelle 3; membranelles arranged in a shape similar to a triangle (Figs. 4D, 5L, 9E). Paroral membrane elongated bracket-shape, commences in mouth pocket at caudal end of membranelle 3, exits mouth pocket posteriorly, extends onto ventral surface, occupies about one-sixth of body length (Figs. 4D, 5L, 9E). One postoral kinety row to left of SK1, starts at level of posterior quarter of paroral membrane, terminates at anterior end of postoral suture (Figs. 4E, 5I).

### *Conchophthirus paracurtus* sp. nov. (Figs. 6, 7; Table 1)

**Fig. 6:**
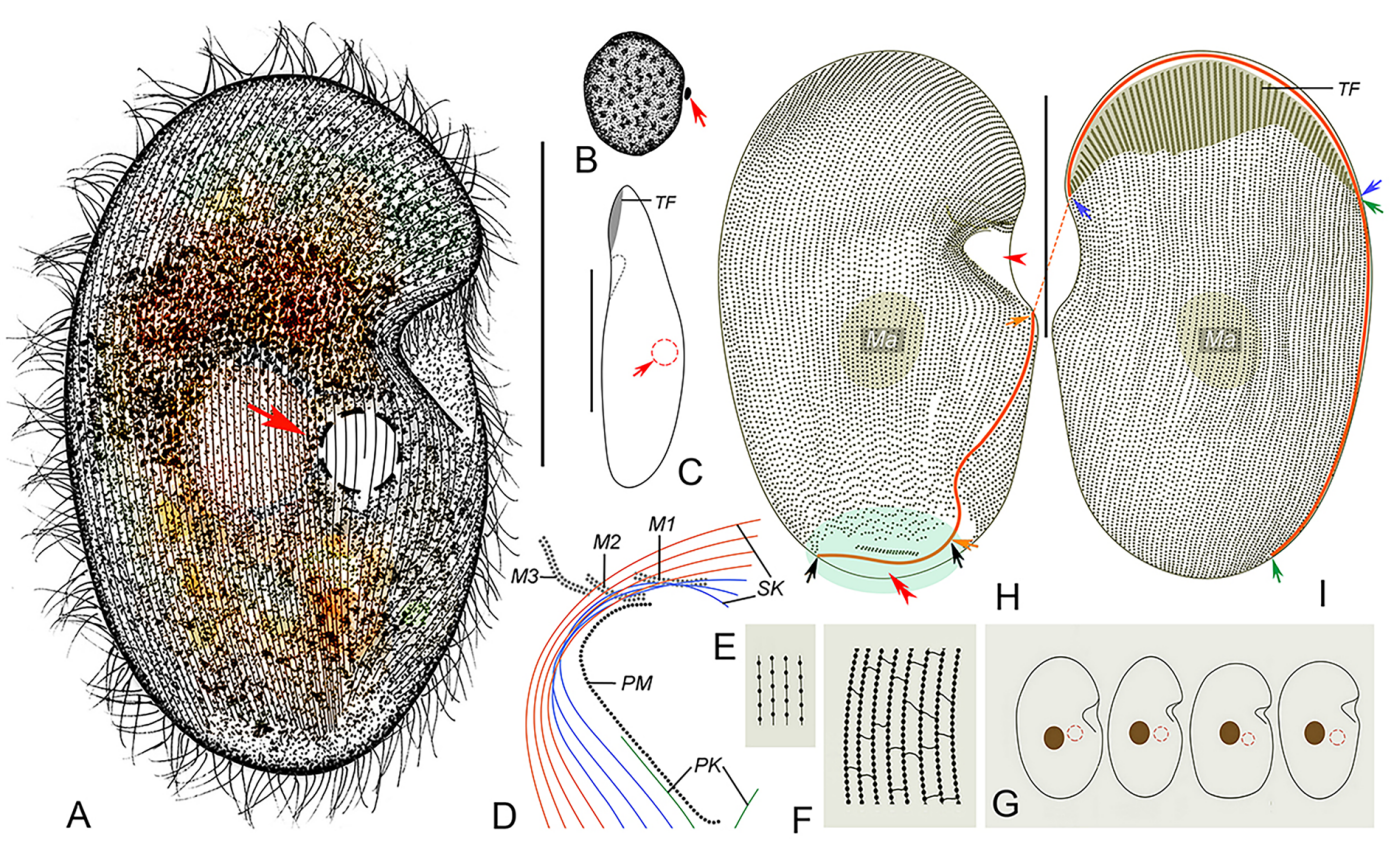
*Conchophthirus paracurtus* sp. nov. in vivo (**A**, **C**, **G**), after protargol (**B**, **D**, **H**, **I**) and silver nitrate (**E**, **F**) staining. **A** Right ventrolateral view of a representative cell, arrow shows the contractile vacuole. **B** Nuclear apparatus, arrow shows the micronucleus. **C** Lateral view, arrow marks the contractile vacuole. **D** Oral apparatus, the blue lines represent the somatic kineties wrapped within the mouth pocket, and the orange lines represent the exposed somatic kineties. **E**, **F** Silverline system for the posterior (**E**) and anterior (**F**) parts of the body. **G** Variation of body shapes. **H**, **I** Right ventrolateral (**H**) and left dorsolateral (**I**) views, to show the ciliature and location of the nuclear apparatus. Blue arrows denote the anterior suture, green arrows mark the right lateral suture, orange arrows mark the postoral suture, black arrow mark a short transverse suture, red arrowhead marks the glabrous area at the buccal field and red double arrowhead indicates the glabrous area at the posterior body end. *M1–3* membranelle 1–3, *Ma* macronucleus, *PK* postoral kinety; *PM* paroral membrane, *SK* somatic kineties, *TF* thigmotactic field. Scale bars = 30 μm (**A**, **C**), 40 μm (**H**, **I**)

**Fig. 7:**
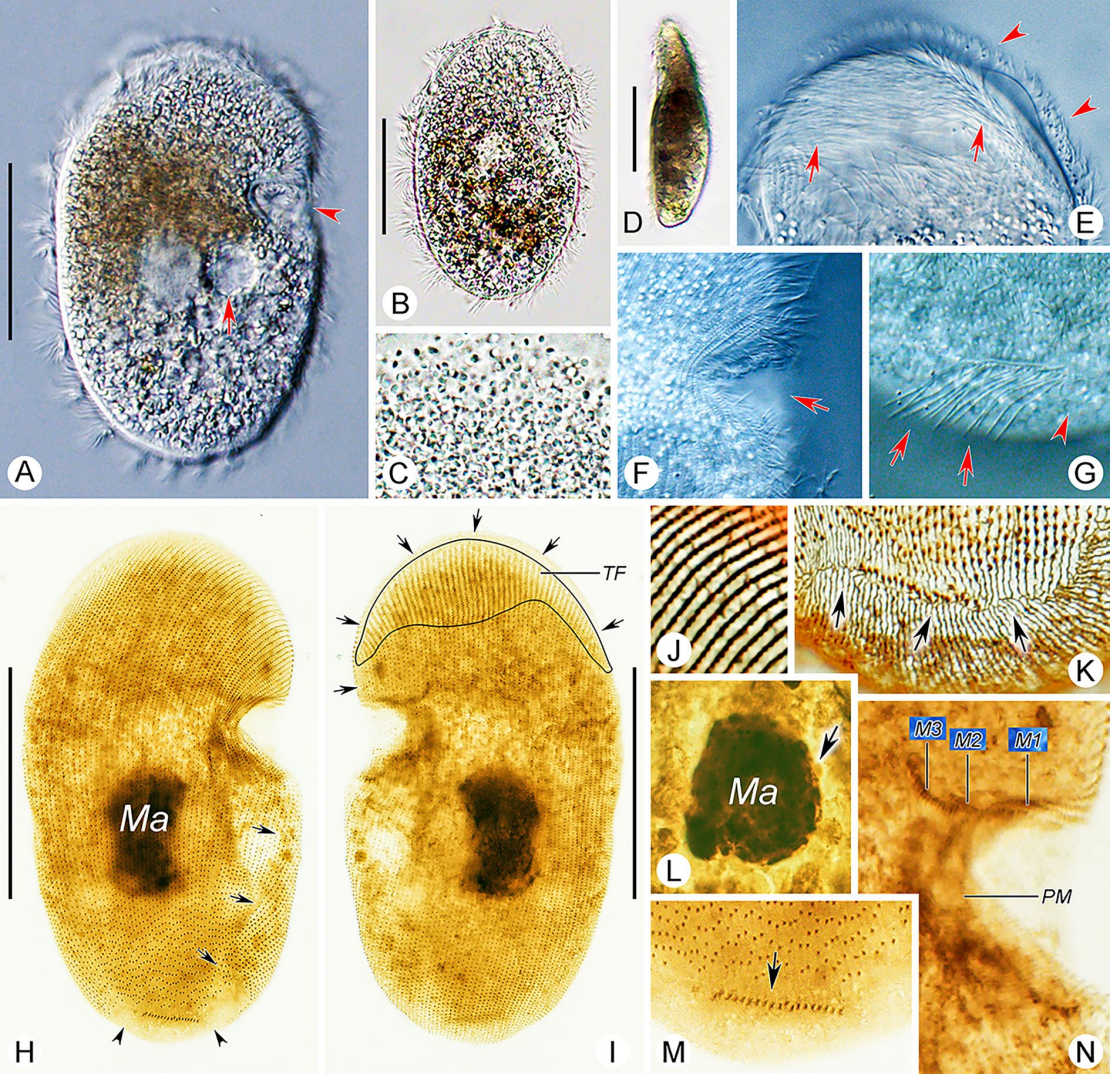
*Conchophthirus paracurtus* sp. nov. in vivo (**A**–**G**), after protargol (**H**, **I**, **L**–**N**) and silver nitrate (**J**, **K**) staining. **A**, **B** Right ventrolateral view of representative cells, arrow in **A** shows contractile vacuole, arrowhead in **A** shows the opening of the mouth pocket. **C** Particles concentrated in the body. **D** Lateral view of a representative cell. **E** Anterior dorsal side of the cell, arrows show the thigmotactic cilia, arrowheads show the somatic cilia. **F** Arrow shows the opening of the mouth pocket. **G** Arrows show the transversely arranged caudal cilia, arrowhead shows the nonciliated area. **H**, **I** Right ventrolateral (**H**) and left dorsolateral (**I**) views of the holotype specimen to show the ciliature, arrows show the suture, arrowheads in **H** show the nonciliated area. **J**, **K** Silverline system for the upper (**J**) and lower (**K**) parts of the body, respectively, arrows in **K** mark the suture-like silverline. **L** Nuclear apparatus, arrow shows the spherical micronucleus. **M** Arrow shows the caudal ciliary row surrounded by the posterior glabrous area. **N** Oral apparatus. *M1–3* membranelle 1–3, *Ma* macronucleus, *PM* paroral membrane, *TF* thigmotactic field. Scale bars = 30 μm (**A**, **B**, **D**), 40 μm (**H**, **I**)

#### Diagnosis

Small *Conchophthirus*, size about 55–75 × 35–45 μm in vivo; body elliptical in outline with both ends broadly rounded; oral apparatus contains three membranelles, each membranelle with two kinety rows; paroral membrane extends from membranelle 2 posteriorly onto ventral surface just below mouth pocket; single macronucleus located at body center, with single adjacent micronucleus; single contractile vacuole located left of midline at cell equator; 96–124 somatic kineties, SK1 to SK3–fivefold into mouth pocket on left cell margin; glabrous area on posterior right ventrolateral surface; about 20–28 caudal cilia arranged in transverse row.

#### Etymology

Composite of the Greek prefix *para-*(beside) and the species-group name *curtus*, referring the similarity between the new species and *Conchophthirus curtus* Engelmann, 1862.

#### Type locality and habitat

Isolated from the mantle cavity and gills of *Cristaria plicata* (Leach, 1814), found in Lake Weishan Wetland (34°45′55′′ N, 117°08′53′′ E), northern China.

#### Deposition of type slide

The protargol-stained slide containing the holotype specimen marked with a red ink circle (Figs. 6H, I, 7H, I) and several paratype specimens marked with black ink circles (registration number: LT2021112401-1) is deposited in the Laboratory of Protozoology, Ocean University of China, Qingdao, China.

#### Gene sequence

The 18S rRNA gene sequence of *Conchophthirus paracurtus* sp. nov. is deposited in GenBank (accession number OR042380). The length and G + C content of the sequence are 1629 bp and 43.16%, respectively. The ITS1-5.8S rRNA-ITS2 region sequence (accession number OR148436) has a length and G + C content of 563 bp and 37.83%, respectively.

#### Description

Body size about 55–75 × 35–45 μm in vivo (Figs. 6A, H, I, 7A, B, H, I). Body slightly variable in shape, elliptical in ventral view, sometimes slightly broader at anterior end (Figs. 6A, G, 7A, B). Length to width ratio about 1.5–2:1. Dorsoventrally flattened about 2–3:1 (Figs. 6C, 7D). Oral apparatus located at junction of anterior and middle thirds on left cell margin, forming a semi-circular notch on left margin (Figs. 6A, 7A, B, F). Cytoplasm colorless, often containing numerous gray granules about 1 μm in diameter, giving cells brown color (Fig. 7A–D). Single contractile vacuole located left of midline and slightly posterior to oral apparatus, about 7–10 µm in diameter in diastole, contracts at about 15 s intervals (Figs. 6A, C, 7A). Single ellipsoidal macronucleus, about 10–15 μm in diameter, located in body center; single ellipsoidal micronucleus, about 2 μm in length, adjacent to macronucleus (Figs. 6B, H, I, 7H, L). No cortical granules or extrusomes observed.

Locomotion usually by gliding at moderate speed with dorsal side attached to substrate, occasionally swims at moderate speed while rotating about long axis.

Somatic cilia about 6–8 μm long, arranged in 96–124 somatic kineties; ventral kineties longitudinally oriented and curved leftward anteriorly; dorsal kineties more or less longitudinal (Figs. 6H, I, 7E, G–I, K, M). Single transverse row of caudal ciliary row composed of about 20–28 dikinetids, surrounded by glabrous area on posterior right ventrolateral surface, caudal cilia about 8–10 μm long (Figs. 6H, 7H). SK1 to SK3–fivefold into mouth pocket (Fig. 6D). All ventral kineties consist of monokinetids; dorsal kineties composed of monokinetids posteriorly and dikinetids anteriorly; arc-shaped thigmotactic field on anterodorsal surface of anterior cell end, occupying about 20% of body length (Figs. 6H, I, 7H, I); thigmotactic cilia about 4–5 µm long in vivo (Fig. 7E). Transverse anterior suture extends from left side of mouth pocket, along ventral–dorsal margin, around anterior end of cell, terminating at same level on right cell margin; postoral suture on ventral surface, obliquely oriented, extending posteriorly from just below mouth pocket to glabrous area (Figs. 6H, I, 7H, I). About 15–20 leftmost dorsal somatic kineties extend around left cell margin onto ventral surface, terminating at postoral suture (Figs. 6H, 7H). Silverline system consisting of prominent primary longitudinal meridians within kineties and inconspicuous irregularly distributed transverse connectives resulting in reticulate pattern of elongated rectangles; ventral primary meridans shortened posteriorly, terminating at transverse caudal ciliary row; dorsal primary meridians curve around posterior pole of cell, terminating at transverse caudal ciliary row (Figs. 6E, F, 7J, K).

Oral apparatus located inside mouth pocket (Figs. 6D, H, 7N, 9E). Three transversely arranged membranelles, each composed of two kinety rows, in more or less increasing order of length (Figs. 6D, 7N, 9E). Paroral membrane bracket-shaped, extending obliquely from membranelle 2 posteriorly onto ventral surface just below mouth pocket (Figs. 6D, 7N). Two postoral kineties, one located on left and one located on right side of paroral membrane (Fig. 6D).

### Phylogenetic analyses (Fig. 8)

**Fig. 8:**
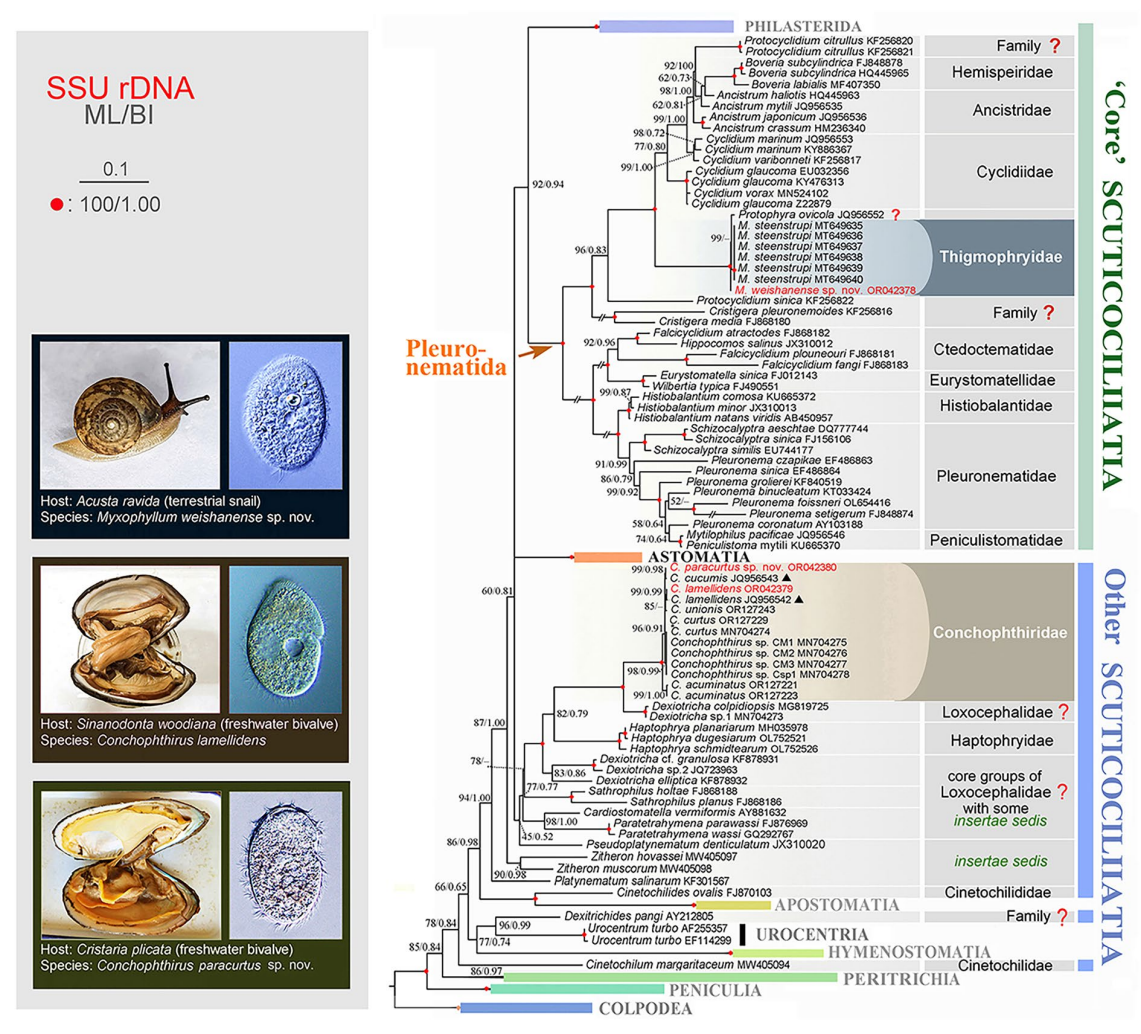
Maximum likelihood (ML) tree inferred from 18S rRNA gene sequences, showing the positions of the newly submitted sequences (in red color). Numbers near branches denote bootstrap values (ML)/Bayesian posterior probabilities (BI). Fully supported (100%/1.00) branches are marked with red solid circles. Black dashes indicate the disagreement between ML and BI trees. Sequences with doubtful identification are marked with triangles. The scale bar corresponds to ten substitutions per 100 nucleotide positions. Double-diagonal slash indicates that the branch length is shortened to fit the width of the figure. Systematic classification mainly follows Lynn (2008) and Gao et al. (2016). Sequences marked with “?”, are not confirmed or the systematic positions are questionable

ML and BI trees based on 18S rRNA gene sequences were constructed to determine the phylogenetic positions of *Myxophyllum weishanense* sp. nov., *Conchophthirus lamellidens*, and *C. paracurtus* sp. nov. Topologies of the ML and BI trees were nearly congruent, hence only the ML tree is shown with branch support from both methods (Fig. 8). *Myxophyllum weishanense* sp. nov. clusters with its congener *M. steenstrupi* (Stein, 1861) Raabe, 1934 and another (possibly misidentified) “thigmotrich” ciliate, namely *Protophyra ovicola* Kofoid, 1903, with full support within the scuticociliate order Pleuronematida. The sequence of “*Protophyra ovicola*” is not associated with published morphological information or a vouchered specimen, therefore its identification is questionable. In addition, the order Pleuronematida is a fully supported monophyletic group that is sister to the scuticociliate order Philasterida with high to moderate support (92% ML and 0.94 BI). The clade consisting of *M. weishanense* sp. nov. + *M. steenstrupi* + so-called *“Protophyra ovicola”* is sister to a highly heterogenous clade encompassing “thigmotrich” ciliates isolated from mollusks and belonging to the genera *Ancistrum* Maupas, 1883 and *Boveria* Stevens, 1901, as well as the free-living cyclidiid species *Protocyclidium citrullus* (Cohn, 1866) Foissner et al., 2002, *Cyclidium glaucoma* Müller, 1773, *C. marinum* Borror, 1963, *C. varibonneti* Song, 2000, and *C. vorax* Pan et al., 2020, with full statistical support.

The sequences obtained from *Conchophthirus lamellidens* and *C. paracurtus* sp. nov. cluster with those from other *Conchophthirus* species and together form a fully supported clade confirming the monophyly of the genus *Conchophthirus*. The *Conchophthirus* clade is placed outside the “core” scuticociliate clade (which consists of the orders Pleuronematida and Philasterida) and nests within the heterogenous cluster of loxocephalid *Dexiotricha* spp. and the mouthless *Haptophrya* spp., with full support. Within the *Conchophthirus* + *Dexiotricha* + *Haptophrya* clade, *Conchophthirus* first group with *Dexiotricha* sp. 1 and *D. colpidiopsis* with full statistical support. The *Conchophthirus* + *Dexiotricha* clade is sister to the genus *Haptophrya* with weak support (82% ML, 0.79 BI). Finally, the *Conchophthirus* + *Dexiotricha* + *Haptophrya* clade together groups with *Dexiotricha* sp. 2, *D. elliptica*, and *Dexiotricha* cf. *granulosa* with full statistical support*.* The separation of *Dexiotricha* taxa among different clades indicates that the genus *Dexiotricha* is paraphyletic.

### Comparisons of the 18S rRNA gene and ITS1-5.8S-ITS2 region sequences (Fig. 9; Table 2)

**Fig. 9:**
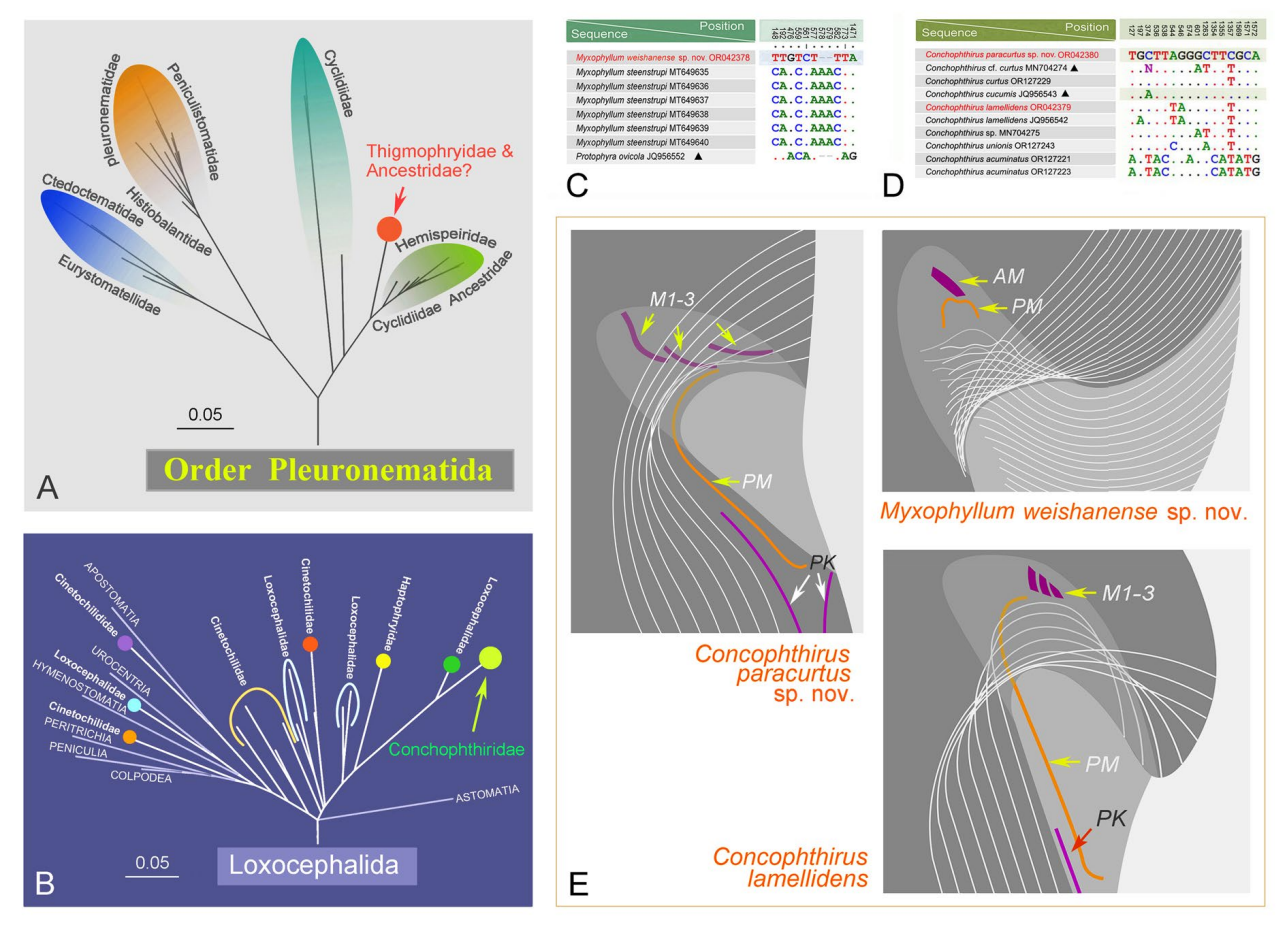
**A**, **B** Relationships among families within Pleuronematida (**A**) and Loxocephalida (**B**), respectively. **C** *Myxophyllum weishanense* sp. nov. OR042378 with related sequences, triangular marker indicates that this sequence may be a misidentification. **D** *Conchophthirus paracurtus* sp. nov. OR042380 and *Conchophthirus lamellidens* OR042379 with related sequences, triangular marker indicates that this sequence may be a misidentification. **E** Details of oral apparatus of *M. weishanense* sp. nov., *C. paracurtus* sp. nov. and *C. lamellidens*. *AM* adoral membranelle, *M1–3* membranelle 1–3, *PM* postoral kineties, *PM* paroral membrane

A BLAST (Basic Local Alignment Search Tool) search of the three newly obtained 18S rRNA gene sequences against the nucleotide NCBI database was carried out to find the most closely related species. The BLASTn algorithm revealed that *Myxophyllum weishanense* sp. nov. (OR042378) is most closely related to its congener *M. steenstrupi* (MT649635‒MT649640) and so-called “*Protophyra ovicola*” (JQ956552). The Weishan population of *Conchophthirus lamellidens* is most closely related to a Qingdao population of *C. lamellidens* (JQ956542) for which no published morphologic information exists. The newly obtained sequence of *Conchophthirus paracurtus* sp. nov. is most closely related to two of its congeners, namely *C. cucumis* (JQ956543) and *C. curtus* (OR127229). The sequence of *M. weishanense* sp. nov. (OR042378) differs from *M. steenstrupi* at seven nucleotide positions and from so-called “*Protophyra ovicola*” at five nucleotide positions (Fig. 9C). The newly obtained sequence of *C. lamellidens* (OR042379) differs from the Qingdao population *C. lamellidens* (JQ956542) at only one nucleotide position and from seven other closely related species at four to 11 nucleotide positions (Fig. 9D). The sequence of *C. paracurtus* sp. nov. (OR042380) is most similar to those of *C. cucumis* (JQ956543) and *C. curtus* (OR127229), differing from each at only one nucleotide position, while it differs from other congeners at more than three nucleotide positions (Fig. 9D).

**Table 2:** Numbers of unmatched nucleotides (above diagonal) and pairwise *p*-distances (below diagonal) of ITS1-5.8S-ITS2 sequences among members of the genus *Conchophthirus*

Species (accession no.)	1	2	3	4	5
1. *C. paracurtus* sp. nov. (OR148436)		85	126	88	101
2. *C. curtus* (OR129888)	0.156		122	44	72
3. *C. lamellidens* (OR148435)	0.222	0.223		107	111
4. *C. unionis* (OR129902)	0.160	0.085	0.198		74
5. *C. acuminatus* (OR129880)	0.184	0.138	0.205	0.142	

Since the 18S rRNA gene sequences of *C. paracurtus* sp. nov. and its closest relatives are strongly conserved, their ITS1-5.8S-ITS2 region sequences are compared. Altogether five sequences are available, including *C. paracurtus* sp. nov. (OR148436), *C. curtus* (OR129888), *C. lamellidens* (OR148435), *C. unionis* (OR129902) and *C. acuminatus* (OR129880). The number of unmatched nucleotide positions and the pairwise *p*-distances among members of the genus *Conchophthirus* are summarized in Table 2. The ITS1-5.8S-ITS2 region sequence divergences between *C. paracurtus* sp. nov. and four other taxa ranged from 15.6% to 22.2%, corresponding to as many as 85–126 unmatched nucleotide positions. The genetic divergence between *C. lamellidens* and four congeners was from 19.8% to 22.3% corresponding to 107 to 126 unmatched nucleotide positions (Table 2).

### Putative internal transcribed spacer 2 secondary structure (Figs. 10, 11)

**Fig. 10:**
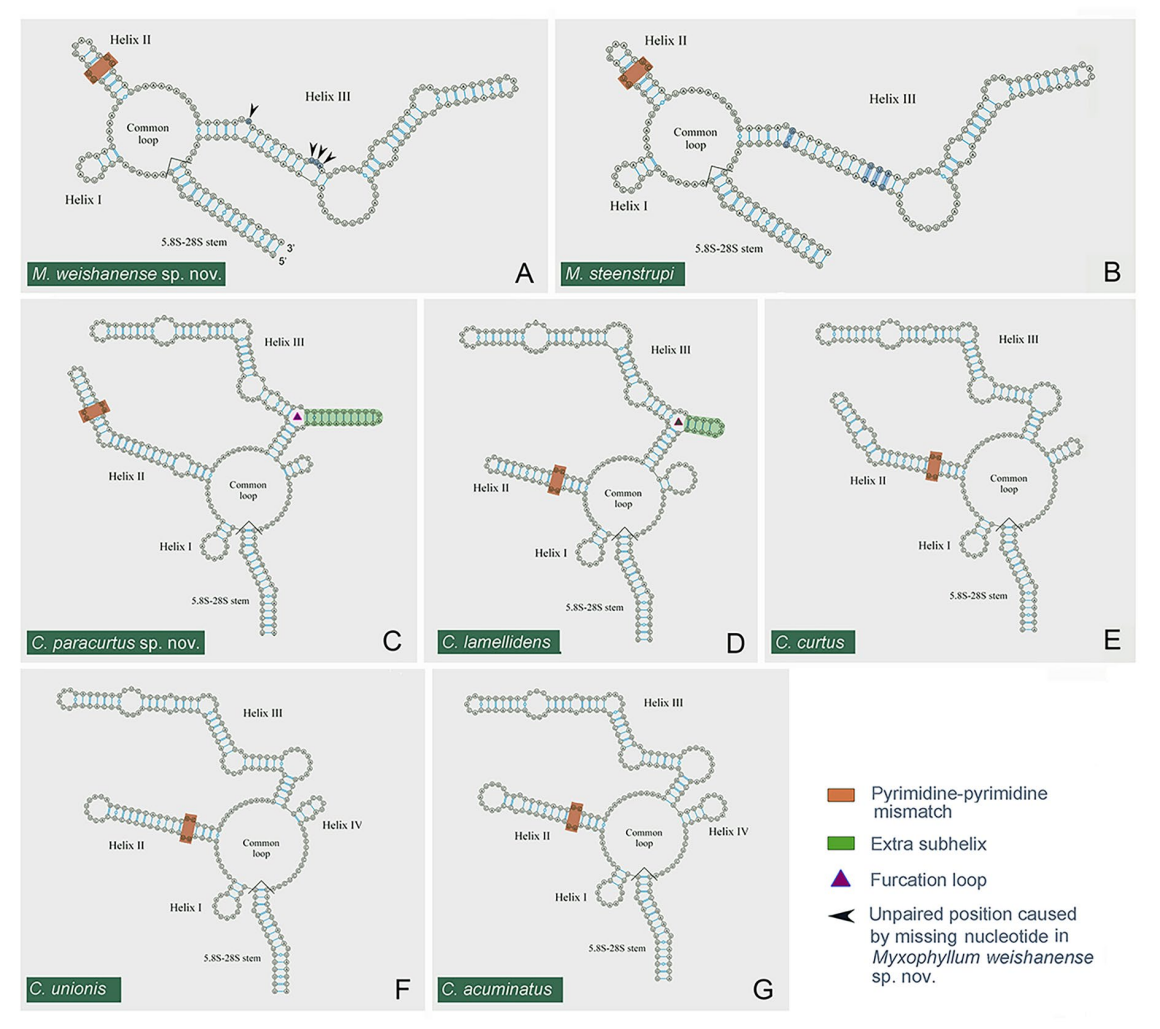
Putative secondary structures of the ITS2 region. **A** *Myxophyllum weishanense* sp. nov. (OR148434). **B** *M. steenstrupi* (Stein, 1861) Raabe, 1934 modified from Zhang and Vďačný (2021). **C** *Conchophthirus paracurtus* sp. nov. (OR148436). **D** *C. lamellidens* (OR148435). **E**–**G** *C. curtus* (OR129888), *C. unionis* (OR129902) and *C. acuminatus* (OR129880) as described in Zhang and Vďačný (2024). Arrowheads mark unpaired nucleotide positions caused deletions in *M. weishanense* sp. nov; triangles represent furcation loop in Helix III

**Fig. 11:**
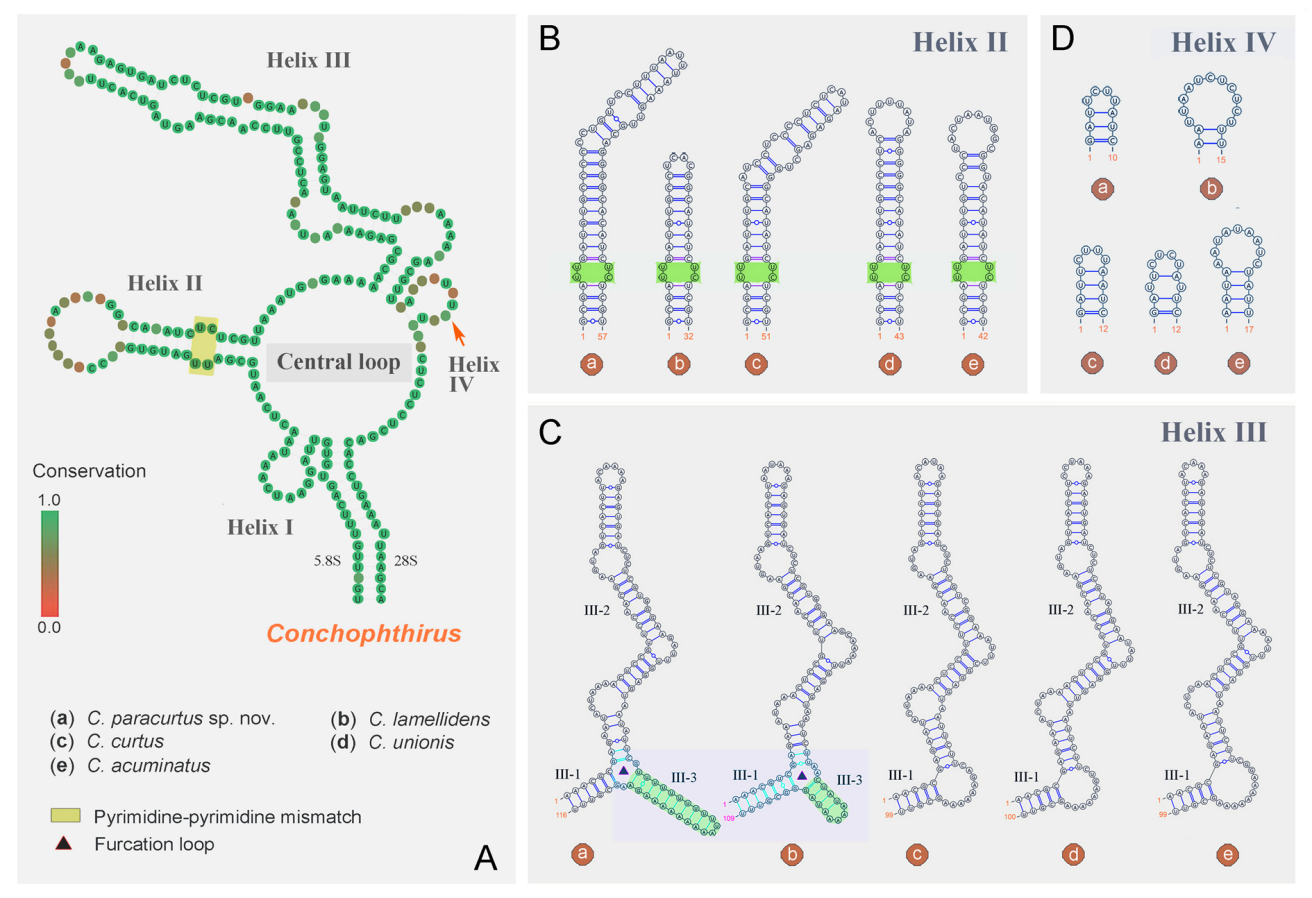
Putative secondary structure models of ITS2 region for the genus *Conchophthirus*. **A** Consensus secondary structure showing a central loop with four helices corresponding to helices I, II, III and IV. Note that helix II has a pyrimidine–pyrimidine mismatch (yellow block). Differences among five species are in helices II–IV. **B**–**D** Different secondary structures of helices II–IV among five species. Green blocks in **B** indicate the pyrimidine–pyrimidine mismatch

The boundaries of the ITS2 region were determined by searching for the 5.8S-28S rRNA hybridization stem with R2DT (Sweeney et al. 2021). There were 15 nucleotide pairs in the hybridized 5.8S-28S rRNA stem. However, Zhang and Vďačný (2021) utilized the putative ITS2 secondary structures of most closely related oligohymenophorean ciliates (Gao et al. 2013; Miao et al. 2008) and used only five pairs to constrain the 5.8S-28S rRNA imperfect helix, which led to an incorrect determination of the 3′-end of the ITS2 region. Here, we considered 15 nucleotide pairs in the 5.8S-28S rRNA hybridization stem as described in Zhang and Vďačný (2024). The length of the ITS2 region is 155 nucleotides in *M. weishanense* sp. nov., 224 nucleotides in *C. paracurtus* sp. nov., and 195 nucleotides in *C. lamellidens*. The predicted secondary structures of the ITS2 region are shown in Figs. 10, 11. In the genus *Myxophyllum,* ITS2 has one central loop model and three helices of unequal length, corresponding to helices I‒III (Fig. 10A, B). The common loop of the *Myxophyllum* ITS2 region consists of 24 nucleotides. Helix I is highly conserved and exhibits a 5′-GUU versus AAC-3′ motif at its stem and a terminal loop of seven nucleotides. Helix II is 20 nucleotides long and exhibits a 5′-GUGG versus CCAU-3′ motif at its stem, a single pyrimidine–pyrimidine bulge consisting of 5′-UU versus UC-3′ (brown blocks in Fig. 10A, B), and a terminal loop of four nucleotides. Helix III is the longest, having as many as 98 nucleotides in *M. weishanense* sp. nov. and 102 nucleotides in *M. steenstrupi*. Helix III of *M. weishanense* sp. nov. is almost identical to that of *M. steenstrupi*, except for four unpaired nucleotides in *M. weishanense* sp. nov. (arrowheads in Fig. 10A).

The secondary structure of *Conchophthirus* ITS2 region is distinctly different from that of *Myxophyllum*. The ITS2 region of the genus *Conchophthirus* possesses not only one central loop model and helices I‒III, but also an additional helix IV (Figs. 10C–G, 11A). The central loop is composed of 26 nucleotides in *C. paracurtus* sp. nov., 24 nucleotides in *C. lamellidens*, 31 nucleotides in *C. curtus*, 30 nucleotides in *C. unionis* and 27 nucleotides in *C. acuminatus* (Fig. 10C–G). Helix I is the shortest and most highly conserved, and exhibits a 5′-UUA versus UAA-3′ motif at its stem and a terminal loop of nine nucleotides, in five *Conchophthirus* species (Fig. 10C–G). Helix II is variable, having as many as 57 nucleotides in *C. paracurtus* sp. nov., 32 nucleotides in *C. lamellidens*, 51 nucleotides in *C. curtus*, 43 nucleotides in *C. unionis* and 42 nucleotides in *C. acuminatus*. (Figs. 10C–G, 11B). Despite this, the five *Conchophthirus* species share one 5′-GCGA versus UCGU-3′ motif at their stems, the same pyrimidine–pyrimidine bulge consisting of 5′-UU versus UC-3′ (brown blocks in Fig. 10C–G and green blocks in Fig. 11B), and six nucleotide pairs composed of 5′-GAUGUG versus CAU(C)AUC-3′. Helix III is the longest and consists of 116 nucleotides in *C. paracurtus* sp. nov., 109 nucleotides in *C. lamellidens*, 99 nucleotides in *C. curtus*, 100 nucleotides in *C. unionis* and 99 nucleotides in *C. acuminatus*. (Figs. 10C–G, 11C). Helix III displays a much more complex structure. In *C. paracurtus* sp. nov. and *C. lamellidens*, it is composed of a furcation loop (triangles in Figs. 10C, D, 11C) and three subhelices, corresponding to subhelices III-1 to III-3, while there are only two subhelices, i.e., III-1 and III-2, in *C. curtus*, *C. unionis* and *C. acuminatus* (Figs. 10C, D, 11C). Subhelix III-3 is highly variable having ten nucleotide pairs in *C. paracurtus* sp. nov. and four nucleotide pairs in *C. lamellidens.* Helix IV is the most variable and differs significantly among the five *Conchophthirus* species. *Conchophthirus paracurtus* sp. nov. exhibits three nucleotide pairs and a terminal loop of four nucleotides, *C. lamellidens* consists of two nucleotide pairs and a terminal loop of 11 nucleotides, *C. curtus* has four nucleotide pairs and a terminal loop of four nucleotides, *C. unionis* is composed of three nucleotide pairs, one bulge and a terminal loop of four nucleotides, and *C. acuminatus* exhibits four nucleotide pairs and a terminal loop of nine nucleotides (Fig. 11D).

## Discussion

### Comparison of *Myxophyllum weishanense* sp. nov. with congeners (Table 3)

**Table 3:** Comparison of *Myxophyllum weishanense* sp. nov. with *M. steenstrupi*

Character	*M. weishanense* sp. nov	*M. steenstrupi* ^a^
Body size (μm)	100–150 × 70–100	120–155 × 90–140
Number of macronuclei	6–8	7–9
Number of SK on ventral side	55–61	87–97
Number of SK on dorsal side	56–62	76–89
Number of VVK	10–17	12–26
Number of DVK	6–10	12–26
Number of thigmotactic kineties	56–62	76–78

*DVK* dorsal vestibular kineties, *SK* somatic kineties, *VVK* ventral vestibular kineties

^a^Data based on the Slovak populations from Zhang and Vďačný (2021)

*Myxophyllum*, which was established as a monotypic genus (type species *M. steenstrupi*), lives mostly in the mantle cavity of terrestrial pulmonate gastropods and has been reported from a variety of hosts from Europe and the USA (de Puytorac et al. 1992; Kazubski 1964, 1973; Penn 1958; Raabe 1971). According to a recent redescription (Zhang and Vďačný 2021), *M. steenstrupi* has the following features: (1) globular to ovoid body; (2) multiple macronuclear nodules grouped in the center of the cell; (3) a contractile vacuole in the mid-region of the body near the group of macronuclei; (4) densely-spaced somatic cilia; (5) oral cavity situated in the left-posterior quarter of the cell; (6) oral apparatus comprising one paroral membrane and one adoral membranelle, both sunken in the buccal cavity.

In previous studies, all *Myxophyllum* isolates have a similar morphology but lack molecular data, and have been identified as *M. steenstrupi,* although some differences are recognizable among such “populations” (e.g., numbers of somatic kineties and macronuclear nodules). In their redescription and review of *M. steenstrupi*, based on populations from Slovakian hosts, Zhang and Vďačný (2021) provided the first 18S rRNA gene sequence for this species. This enabled our new species to be compared with *M. steenstrupi,* mainly represented by the Slovak population.

The new species was isolated from a terrestrial snail, *Acusta ravida,* collected from the Weishan Wetland, China. It differs from the Slovak population of *M. steenstrupi* in two features: (1) fewer somatic kineties (55–61 vs. 87–97 on the ventral side and 56–62 vs. 76–89 on the dorsal side); (2) caudal cilia (conspicuous vs. absent/not mentioned in *M. steenstrupi*). In terms of molecular data, comparison of the 18S rRNA gene sequences reveals seven unmatched nucleotide positions, which supports the distinctness of the two taxa and supports the validity of *Myxophyllum weishanense* sp. nov. as a new species.

In addition, there are about 50 ventral kineties and 55 dorsal kineties in a Polish population (Raabe 1971) and 60 ventral kineties in a French population (Puytorac et al. 1992). Although the number of ventral and dorsal kineties in these populations overlap with *M. weishanense* (55–61 ventral kineties, 56–62 dorsal kineties), these populations lack molecular data and were collected far away from the type locality of *M. weishanense* sp. nov. (Europe vs. China). We do not therefore consider them to be conspecific with *M. weishanense* sp. nov. pending further data for these populations.

### Remarks on the genus *Conchophthirus*

Ehrenberg (1838) first recorded a *Conchophthirus* species in the mantle cavity of bivalves, under the name *Leucophrys anodontae*. Subsequently, Stein (1861) established the currently accepted genus *Conchophthirus* with *L. anodontae* as the type species. Since then, many *Conchophthirus* species have been reported from all over the world (Engelmann 1862; Ghosh 1918; Raabe 1933, 1934, 1971). There have been many synonyms and misidentifications as detailed by Raabe (1971) in his monograph on *Conchophthirus*. According to Raabe’s (1971) revision, *Conchophthirus* included ten valid species: *C. anodontae* (Ehrenberg, 1838) Stein, 1861, *C. lamellidens* Ghosh, 1918, *C. elongatus* Ghosh, 1918, *C. unionis* Raabe, 1932, *C. cucumis* Uyemura, 1935, *C. curtus* Engelmann, 1862, *C. discophorus* Mennod, 1914, *C. acuminatus* (Claparede & Lachmann, 1858) Raabe, 1933, *C. klimentinus* Raabe, 1965, and *C. magna* Kidder, 1934. In the same year, Antipa and Small (1971a) made a complete morphological redescription of *C. curtus*. Unfortunately, the details of the oral ciliature, a crucial taxonomic feature, are lacking for most other species.

### Comparison of *Conchophthirus paracurtus* sp. nov. with its most morphologically similar congeners (Table 4)

**Table 4:** Comparison of *Conchophthirus paracurtus* sp. nov. with closely related congeners

Species	Data source	Body size in vivo (μm)	Average size (μm)	Nonciliated area	SK, no.	Folded SK, no.	Kineties in M1–3, no.
*C. paracurtus* sp. nov	Present study	55–75 × 35–45	66 × 40	Present	96–124	3–5	2, 2, 2
*C. curtus*	Raabe (1971)	60–150 × 50–100	120 × 70	Present	150–160	3–5	3, 3, 3
*C. unionis*	Raabe (1971)	80–170 × 30–100	120 × 60	Absent	90–95	6	NA
*C. klimentinus*	Raabe (1971)	60–130 × 40–100	101 × 55	Absent	160	Several	NA
*C discophoru*s	Raabe (1971)	60–110 × 60–100	90 × 85	Absent	150	Several	NA
C. *magna*	Raabe (1971)	123–203 × 63–116	180 × 95	Absent	275	1–3	NA

*M1–3* membranelles 1–3, *NA* not available, *No.* number, *SK* somatic kineties

Based on its body shape and general morphology, *Conchophthirus paracurtus* sp. nov. should be compared with five congeners: *C. curtus*, *C. unionis*, *C. klimentinus*, *C. discophoru*s, and *C. magna*.

Morphologically, four of these, i.e., *Conchophthirus unionis*, *C. klimentinus*, *C. discophoru*s, and *C. magna* can be easily distinguished from *C. paracurtus* sp. nov. by the absence of a glabrous right posterolateral area (vs. present in *C. paracurtus*), and body size (*C. paracurtus* is larger than all four) (Raabe 1971). *Conchophthirus curtus* is morphologically most similar to *C. paracurtus* sp. nov. Both species are similar in body outline, with a nonciliated area surrounding a transverse row of about 20–30 caudal cilia on the right posterior ventrolateral side (Antipa and Small 1971a; Raabe 1971; Zhang and Vďačný 2024). However, *C. paracurtus* sp. nov. has a smaller body size (55–75 × 35–45 μm vs. 60–150 × 50–100 μm) and fewer somatic kineties (96–124 vs. 150–160), clearly distinguishing it from *C. curtus*.

Although the 18S rRNA gene sequence of *C. paracurtus* sp. nov. (OR042380) differs from that of *C. curtus* (OR127229) at only one nucleotide position, its ITS1-5.8S-ITS2 region sequence differs substantially from that of *C. curtus*, i.e., the genetic divergence is 15.6% (85 unmatched nucleotide positions), and the secondary structure of the ITS2 region for *C. paracurtus* sp. nov. is distinctly different from that of *C. curtu*s (e.g., having the extra subhelix III-3 in Helix III as shown in Fig. 11C). Taken together, the morphologic and genetic differences support the validity of *C. paracurtus* as a new species.

### Identification of *Conchophthirus lamellidens* Ghosh, 1918 and comparison with related species (Table 5)

**Table 5:** Comparison of *Conchophthirus lamellidens* with closely related congeners

Species	Data source	Body size in vivo (μm)	Average size (μm)	Caudal bulge	SK, no.	Folded SK, no.
*C. lamellidens*	Present study	80–110 × 35–65	94 × 56	Present	62–74	7–9
*C. acuminatus*	Raabe (1971)	50–120 × 40–60	100 × 50	Absent	90	10
*C. anodontae*	Raabe (1971)	80–170 × 40–120	120 × 70	Absent	80	10–15
*C. cucumis*	Raabe (1971)	87–141 × 45–72	112 × 60	Absent	NA	NA

*No.* number, *NA* not available, *SK* somatic kineties

In the original description of *C. lamellidens* (Gosh 1918), the mouth pocket is located on the right margin of the ventral surface, opposite to not only other *Conchophthirus* taxa but to almost all ‘typical’ thigmotrichs in which the mouth pocket is on the left margin of the ventral surface (Kahl 1931). Thus it is likely that Gosh (1918) drew a cell oriented “ventral side down” (i.e., with the dorsal surface toward the viewer). On the other hand, Uyemura (1935) rediscovered *C. lamellidens* in Japan and briefly redescribed the population based on some in vivo data, i.e., body size and shape, the position of the nuclear apparatus, contractile vacuoles, the entrance to the buccal cavity and host locality (Raabe 1971). The following characteristics of the Lake Weishan population of *C. lamellidens* overlap with that described by Uyemura (1935): body size (80–100 × 45–65 μm vs. 90–108 × 35–54 μm), number of vestibular kineties (7–9 vs. about 7), body shape, position of the nuclear apparatus, position of the contractile vacuole, and location and morphology of the buccal cavity. In addition, these two populations share similar hosts (*Sinanodonta* species) and host localities (China vs. Japan). Additionally, one published 18S rRNA gene sequence (JQ956542) marked with the name *C. lamellidens* was found in GenBank. It was collected in Qingdao, China, many years ago, but was not associated with morphological information. The 18S rRNA gene sequence of the Lake Weishan population of *C. lamellidens* differs at only one nucleotide position from the Qingdao population, so we consider these to be conspecific. For elucidating the relationships among *Conchophthirus* species, taxon sampling must be increased and a broader range of marker genes should be analysed.

Based on its living morphology and infraciliature, *C. lamellidens* should be compared with three congeners, namely *C. acuminatus*, *C. anodontae*, and *C. cucumis*. *Conchophthirus lamellidens* has a posterior ventral bulge, which is absent in the other three species. In addition, *C. lamellidens* has fewer somatic kineties compared to *C. acuminatus* (62–74 vs. 90) and has a smaller body size compared to *C. anodontae* (80–100 × 45–65 μm vs. 80–170 × 40–120 μm). Our newly obtained 18S rRNA gene sequence from the Weishan population *C. lamellidens* (OR042379) differs from seven closely related species at four to eleven nucleotide positions (Fig. 9D).

### Phylogenetic positions of *Myxophyllum* and *Conchophthirus* (Figs. 8, 9A, B)

The genus *Myxophyllum* was first described by Stein (1861). The only species of *Myxophyllum*, *M. steenstrupi*, originally belonged to *Conchophthirus.* Owing to significant differences in the oral ciliature, however, Raabe (1934) established the genus *Myxophyllum* with *Conchophthirus steenstrupi* as type species. Raabe (1971) assigned *Myxophyllum* to the family Thigmophryidae, order Thigmotrichida. The family was placed in the order Philasterida in the subclass Scuticociliatia, because of its short paroral membrane (Lynn and Small 2002; Small and Lynn 1985). Lynn (2008) went on to classify *Myxophyllum* in the family Thigmophryidae, order Philasterida, subclass Scuticociliatia.

Zhang and Vďačný (2021) first analysed the phylogenetic position of *Myxophyllum* based on the molecular data of the 18S, 5.8S, 16S, and 28S regions of the ribosomal RNA gene and the mitochondrial cytochrome *c* oxidase I gene. Phylogenetic analysis strongly rejected the inclusion of *Myxophyllum* in the order Philasterida, and fully supported *Myxophyllum* as belonging to the order Pleuronematida. Furthermore, Zhang and Vďačný (2021, 2023b) proposed to transfer all members of the order Thigmotrichida into the order Pleuronematida, and proposed possible reasons for the divergence of *Myxophyllum* from typical pleuronematids, i.e., that the symbiotic lifestyle of *Myxophyllum* is likely responsible for the dramatic remodelling of its oral apparatus and ciliature (e.g., the transfer of the mouth pocket to the posterior body region, the strong reduction of the paroral membrane and adoral organelles, and the formation of vestibular kineties). The general topology of the 18S rRNA gene phylogenetic tree in the present study is consistent with previously published phylogenetic studies (Antipa et al. 2016, 2020; Gao et al. 2013; Poláková et al. 2021; Rataj and Vďačný 2018, 2022; Zhang and Vďačný 2024), and the phylogenetic position of *Myxophyllum* is consistent with Zhang and Vďačný (2021), that is *Myxophyllum* belongs to the family Thigmophryidae, in the order Pleuronematida.

Hitherto, a total 43 18S rRNA gene sequences of *Conchophthirus* were available in GenBank, including two unpublished sequences from Chinese populations, i.e., *C. cucumis* (JQ956543) and *C. lamellidens* (JQ956542), five published sequences from an American population of *Conchophthirus* cf. *curtus* (MN704274), the uncertain species (MN704275-8) in Antipa et al. (2020), and 36 published sequences from three species, i.e., *C. acuminatus* (OR127221–8), *C. curtus* (OR127229-42) and *C. unionis* (OR127243-56).

The present 18S rRNA gene phylogenetic analysis shows that all *Conchophthirus* sequences cluster together as a monophyletic group with full statistical support and are nested deep within the genus *Dexiotricha* with very strong support (99% ML, 1.00 BI), which is consistent with the previous studies (Antipa et al. 2020; Poláková et al. 2021; Zhang and Vďačný 2024). Within this group, the *Conchophthirus* clade clusters with the *D. colpidiopsis* (MG819725) + *Dexiotricha* sp.1 (MN704273) clade. *Dexiotricha colpidiopsis* (MG819725) was reported by Qu et al. (2019) and its ciliature has a typical *Dexiotricha*-pattern, which is distinctly different from that of *Conchophthirus*. However, the longitudinal cortical ridge microtubule of *C. curtus* is in the identical location as that of *D. colpidiopsis* (Antipa 1971, 2014; Peck 1977), which might be regarded as an ultrastructural feature that is common to both clades, thus explaining their close relationship. Other *Dexiotricha* species that are not clustered with *D. colpidiopsis* might have a different ultrastructure, therefore further studies are needed to provide insights into the polyphyly of *Dexiotricha*.

### Host range of *Conchophthirus* (Supplementary Table S2)

*Conchophthirus* species live in the mantle cavity or on the gill surface in various freshwater bivalves, mainly the unionids (Antipa et al. 2020; Grizzle and Brunner 2007; Raabe 1971; Zhang and Vďačný 2024). They usually swim freely within the mantle cavity and are not firmly attached to host tissues. If removed from their hosts, they usually die within 24 h (Kidder 1934; present study). They are currently considered to be endocommensals but possible pathological effects have not been adequately evaluated. They probably feed on epithelial cells shed by their host. *Conchophthirus* species are widely distributed and have been detected in many countries in Asia (Deshmukh et al. 2011; Kahl 1931; Uyemura 1935), Europe (Burlakova et al. 1998, 2006; Chuševė et al. 2012; Conn et al. 2008; Dobrzańska 1958; Fenchel 1965, 1966; Fokin et al. 2003; Karatayev and Burlakova 2022; Karatayev et al. 2000a, b, 2007; Laruelle et al. 1999; Mastitsky 2012; Mastitsky et al. 2008; Mermod 1914; Molloy et al. 2010; Raabe 1933, 1934, 1950, 1966, 1971; Zhang and Vďačný 2024) and North America (Antipa and Hatzidimitriou 1981; Antipa and Small 1971a, b; Antipa et al. 2020; Kidder 1934; Laruelle et al. 2002; Penn 1958). In addition, *Conchophthirus* species inhabit a diverse array of freshwater bivalve groups. Their host range was thoroughly reviewed by Raabe (1971) and by Grizzle and Brunner (2007). According to their reviews, their host range includes more than 34 species belonging to 19 genera of three families and one order (Dreissenidae Gray, 1840, Sphaeriidae Deshayes, 1855 and Unionida Gray, 1854: Supplementary Table S2).

Examination of the ten valid *Conchophthirus* species reveals different correlations between individual species and their host organisms. Some species possibly have “universal occurrence” in unionids and these species also show changes in host preferences according to the geographical region. For example, the most commonly studied species, *C. curtus*, is cosmopolitan and inhabits *Anodonta cygnea* and *Unio* species in Europe, *Sinanodonta lauta* in Japan, and various host species belonging to 13 genera in the USA (Supplementary Table S2). All of these host species belong to the family Unionidae Rafinesque, 1820. Moreover, *C. anodontae* and *C. unionis* share the same host species as *C. curtus* and may coexist in a single host, although their abundance may differ in various locations in the mantle cavity (Antipa et al. 2020; Fenchel 1965; Kidder 1934). *Conchophthirus magna* has only been reported once in the USA and was found to coexist with *C. anodontae* in a single host (about 10–20 individuals in one host specimen) (Kidder 1934). On the other hand, *C. acuminatus*, *C. discophorus*, and *C. klimentinus* appear to show a higher degree of host specificity. *Conchophthirus acuminatus* and *C. klimentinus* have so far been reported only from *Dreissena polymorpha* of the family Dreissenidae, and *C. discophorus* only from members of the family Sphaeriidae. Three *Conchophthirus* species, namely, *C. cucumis*, *C. elongatus*, and *C. lamellidens* from India and Japan, were incompletely described due to the lack of silver stained specimens, hence taxon sampling must be increased and detailed morphological data based on the modern methods, together with molecular data from additional marker genes, are required to verify the reliability of identifications.

The findings of the present study reveals a broader host range for *Conchophthirus* than had previously been documented, including one new species *C. paracurtus* sp. nov. from the Chinese pond mussel *Sinanodonta woodiana* (Lea, 1834) and *C. lamellidens* from the pearl mussel *Cristaria plicata* (Leach, 1815). It is generally believed that the majority of endocommensal ciliates are not restricted to a single host species, which also appears to be the case for most *Conchophthirus* species. Therefore, a broader range of potential host organisms needs to be investigated.

### Supplementary Information

Below is the link to the electronic supplementary material.

Supplementary file 1 (DOCX 40 kb)

## Data Availability

All data generated or analysed during this study can be found in online repositories. The names of the repositories and accession numbers can be found at: https://www.ncbi.nlm.nih.gov/genbank/.
