# Supplementary material for: Findings on three endocommensal scuticociliates (Protista, Ciliophora) from freshwater mollusks, including their morphology and molecular phylogeny with descriptions of two new species
Source: Mar Life Sci Technol. 2024 May 21;6(2):212–35. doi: 10.1007/s42995-024-00230-4 (PMC11136938; doi:10.1007/s42995-024-00230-4)
Supplement: Supplementary file 1 — (DOCX 40 kb) [file 42995_2024_230_MOESM1_ESM.docx]

**Supplementary materials**

**Findings on three endocommensal scuticociliates (Protista, Ciliophora) from freshwater mollusks including their morphology and molecular phylogeny with descriptions of two new species**

**Tao Li^1*^, Tengyue Zhang^2*^, Mingjian Liu^1,3*^, Zhe Zhang^1^, Jiachen Zhang^1^, Junhua Niu^1^, Xiangrui Chen^4^, Saleh A. Al-Farraj^5^, Weibo Song^1,6**^**

^1^ Institute of Evolution & Marine Biodiversity, Ocean University of China & Key Laboratory of Evolution & Marine Biodiversity (Ministry of Education), Qingdao 266003, China

^2^ The Key Laboratory of Zoological Systematics and Application, College of Life Sciences, Hebei University, Baoding 071002, China

^3^ College of Marine Life Sciences, Ocean University of China, Qingdao 266003, China

^4^ School of Marine Sciences, Ningbo University, Ningbo 315800, China

^5^ Zoology Department, College of Science, King Saud University, Riyadh 11451, Saudi Arabia

^6^ Laboratory for Marine Biology and Biotechnology, Laoshan Laboratory, Qingdao 266237, China

*These authors contributed equally to this work.

**Corresponding authors: E-mail: wsong@ouc.edu.cn

Pages: 7

Tables: 2

**Supplementary Table S1** **|** List of taxa with GenBank accession numbers of corresponding 18S rRNA gene sequences, newly sequenced species are in bold.

| **Taxon** | **18S rRNA gene** | **Taxon** | **18S rRNA gene** |
| --- | --- | --- | --- |
| *Almophrya bivacuolata* | HQ446281 | *Gymnodinioides pitelkae* | EU503534 |
| *Ancistrum crassum* | JQ956538 | *Haptophrya dugesiarum* | OL752521 |
| *Anoplophrya lumbrici* | MN121062 | *Haptophrya planariarum* | MH035978 |
| *Anoplophrya marylandensis* | AY547546 | *Haptophrya schmidtearum* | OL752526 |
| *Apofrontonia dohrni* | AM072621 | *Hippocomos salinus* | JX310012 |
| *Astylozoon enriquesi* | AY049000 | *Histiobalantium minor* | JX310013 |
| *Boveria subcylindrica* | HQ445965 | *Homalogastra setosa* | EF158848 |
| *Campanella umbellaria* | AF401524 | *Hyalophysa bradburyae* | MN537438 |
| *Cardiostomatella vermiformis* | AY881632 | *Hyalophysa chattoni* | EU503536 |
| *Cinetochilides ovalis* | FJ870103 | *Hyalophysa lwoffi* | EU503538 |
| *Clausilocola apostropha* | MZ825342 | *Lembadion bullinum* | AF255358 |
| *Colpoda lucida* | EU039895 | *Maryna umbrellata* | JF747217 |
| *Colpoda magna* | EU039896 | *Mesanophrys carcini* | JN885085 |
| *Conchophthirus* cf*. curtus* | MN704274 | *Metacollinia luciensis* | MH200620 |
| *Conchophthirus cucumis* | JQ956543 | *Metaracoelophrya intermedia* | HQ446278 |
| *Conchophthirus lamellidens* | JQ956542 | *Metaradiophrya lumbrici* | MN121071 |
| *Conchophthirus* sp. CM1 | MN704275 | *Metaradiophrya sp.* | HQ446279 |
| *Conchophthirus acuminatus* JR 40 DP | OR127221 | *Metaradiophrya varians* | MN121076 |
| *Conchophthirus acuminatus* VO 161 DB | OR127223 | *Hinoftirius magnum* | JQ956547 |
| *Conchophthirus curtus* HO 30 UC | OR127229 | *Myxophyllum steenstrupi* | MT649635-40 |
| *Conchophthirus unionis* HO 24 UC | OR127243 | ***Myxophyllum weishanense*** | **OR042378** |
| ***Conchophthirus paracurtus*** | **OR042380** | *Njinella prolifera* | HQ446276 |
| ***Conchophthirus lamellidens*** | **OR042379** | *Ophrydium versatile* | AF401526 |
| *Cristigera media* | FJ868180 | *Paraclausilocola constricta* | HQ446275 |
| *Cyclidium marinum* | JQ956553 | *Paraclausilocola elongata* | HQ446274 |
| *Dexiostoma campylum* | X56532 | *Paramecium duboscqui* | AF100312 |
| *Dexiostoma sabulum* | KY218611 | *Paramecium tetraurelia* | X03772 |
| *Dexiostoma saccharum* | KY218610 | *Paranophrys magna* | JN885089 |
| *Dexiotricha cf. granulosa* | KF878931 | *Paratetrahymena wassi* | GQ292767 |
| *Dexiotricha colpidiopsis* | MG819725 | *Philaster apodigitiformis* | FJ648350 |
| *Dexiotricha elliptica* | KF878932 | *Pilasterides armatalis* | FJ848877 |
| *Dexiotricha* sp.1 | MN704273 | *Plagiopyliella pacifica* | AY541685 |
| *Dexiotricha* sp.2 | JQ723963 | *Platyophrya bromelicola* | EU039906 |
| *Dexitrichides pangi* | AY212805 | *Pleuronema wiackowskii* | JX310016 |
| *Durchoniella brasili* | FN998990 | *Porpostoma notata* | HM236335 |
| *Durchoniella legeriduboscqui* | FN998995 | *Protophyra ovicola* | JQ956552 |
| *Entodiscus borealis* | AY541687 | *Pseudocohnilembus marinus* | Z22880 |
| *Entorhipidium tenue* | AY541688 | *Pseudocollinia brintoni* | HQ591470 |
| *Epicarchesium abrae* | DQ190462 | *Pseudocollinia oregonensis* | HQ591473 |
| *Epistylis plicatilis* | AF335517 | *Pseudocollinia similis* | HQ591478 |
| *Eudrilophrya complanata* | HQ446280 | *Pseudoplatynematum denticulatum* | JX310020 |
| *Falcicyclidium atractodes* | FJ868182 | *Pseudovorticella punctata* | DQ190466 |
| *Frontonia didieri* | DQ885986 |  |  |
| *Frontonia vernalis* | U97110 | *Sathrophilus holtae* | FJ868188 |
| *Fusiforma themisticola* | KF516511 | *Telotrochidium matiense* | EF417835 |
| *Glaucoma chattoni* | X56533 | *Tetrahymena foissneri* | MW827176 |
| *Glaucoma* sp. | KY218621 | *Tetrahymena unionis* | MW827181 |
| *Glauconema trihymene* | GQ214552 | *Tetrahymena acanthophora* | MN994469 |
| *Tetrahymena dugesiae* | MK454732 | *Urceolaria mitra* | MW759668 |
| *Tetrahymena malaccensis* | M26360 | *Urceolaria parakorschelti* | KP698204 |
| *Tetrahymena nigricans* | MN994472 | *Urceolaria urechi* | FJ499388 |
| *Tetrahymena paravorax* | EF070253 | *Urocentrum turbo* | AF255357 |
| *Tetrahymena pigmentosa* | M26358 | *Uronema elegans* | AY103190 |
| *Tetrahymena pyriformis* | EF070254 | *Uronemella filificum* | EF486866 |
| *Tetrahymena scolopax* | KJ028504 | *Vaginicola crystallina* | AF401524 |
| *Tetrahymena thermophila* | MH051926 | *Vampyrophrya pelagica* | EU503539 |
| *Trichodina polycelis* | MW759641 | *Wilbertia typica* | GFJ490551 |
| *Trichodina unionis* | ON970917 | *Zoothamnium pluma* | DQ662854 |

**Supplementary Table S2** **|** List of hosts of *Conchophthirus* spp.

| **Host species^a^** | | **Location** | **Reference^b^** |
| --- | --- | --- | --- |
| **Hosts of *C. anodontae* (Ehrenberg, 1938) Stein, 1961** | |  |  |
| *Anodonta californiensis* (Lea, 1852) **[U]** | California, U.S.A. | Antipa et al. (2020) |  |
| *Anodonta cygnea* (Linnaeus, 1758) **[U]** | Poland; Hungary; Bulgaria; Copenhagen, Denmark | Raabe (1933); Fenchel (1965, 1966) |  |
| *Elliptio complanatus* (=*Elliptio complanata* ([Lightfoot], 1786)) **[U]** | Woods Hole, Massachusetts, U.S.A. | Kidder (1934) |  |
| *Margaritifera falcata* (Gould, 1850) **[U]** | California, U.S.A. | Antipa et al. (2020) |  |
| **Hosts of *C. lamellidens* Ghosh, 1918** | |  |  |
| *Anodonta lauta* (=*Sinanodonta lauta* (Martens, 1877) **[U]** | Saitama, Japan | Uyemura (1935) |  |
| *Lamellidens* sp. **[U]** | India | Kahl (1931) |  |
| *Sinanodonta woodiana* (Lea, 1834) **[U]** | Lake Weishan, Shandong Province, China | present study |  |
| **Host of *C. elongatus* Ghosh, 1918** | |  |  |
| *Lamellidens* sp. **[U]** | India | Kahl (1931) |  |
| **Hosts of *C. unionis* Raabe, 1932** | |  |  |
| *Anodonta cygnea* (Linnaeus, 1758) **[U]** | Poland; Lake Balaton, Hungary; Bulgaria; Lake Ohrid, Yugoslavia | Raabe (1971) |  |
| *Sinanodonta woodiana* (Martens, 1877) **[U]** | | Slovakia | Zhang and Vďačný (2024) |
| *Unio crassus* Philipsson, 1788 **[U]** | Poland; Lake Balaton, Hungary; Bulgaria; Lake Ohrid, Yugoslavia, Slovakia | Raabe (1971); Zhang and Vďačný (2024) |  |
| *Unio pictorum* (Linnaeus, 1758) **[U]** | Poland; Lake Balaton, Hungary; Bulgaria; Lake Ohrid, Yugoslavia | Raabe (1971) |  |
| *Unio tumidus* Philipsson, 1788 **[U]** | Poland; Lake Balaton, Hungary; Bulgaria; Lake Ohrid, Yugoslavia | Raabe (1971) |  |
| **Host of *C. cucumis* Uyemura, 1935** | |  |  |
| *Anodonta lauta* (=*Sinanodonta lauta* (Martens, 1877)) **[U]** | Saitama, Japan | Uyemura (1935) |  |
| **Hosts of *C. curtus* Engelmann, 1862** | |  |  |
| *Actinonaias carinata* (=*Ortmanniana ligamentina* (Lamarck, 1819)) **[U]** | Illinois, U.S.A. | Antipa and Small (1971) |  |
| *Alasmidonta marginata* Say, 1818 **[U]** | Woods Hole, Massachusetts; Lake Chautauqua, New York; IL, U.S.A. | Kidder (1934); Antipa and Small (1971) |  |
| *Alasmidonta undulata* (Say, 1817) **[U]** | Woods Hole, Massachusetts; Lake Chautauqua, New York, U.S.A. | Kidder (1934) |  |
| *Anodonta californiensis* Lea, 1852 **[U]** | California, U.S.A. | Antipa et al. (2020) |  |
| *Anodonta cataracta* (=*Pyganodon cataracta* (Say, 1817)) **[U]** | Woods Hole, Massachusetts; Lake Chautauqua, New York, U.S.A. | Kidder (1934) |  |
| *Anodonta cygnea* (Linnaeus, 1758) **[U]** | Poland; Hungary; Bulgaria; Lake Ohrid, Yugoslavia | Raabe (1971) |  |
| *Anodonta grandis* (=*Pyganodon grandis* (Say, 1829)) **[U]** | Iowa; Illinois, U.S.A. | Penn (1958); Antipa (1971); Antipa and Small (1971) |  |
| *Anodonta lauta* (=*Sinanodonta lauta* (Martens, 1877)) **[U]** | Japan | Uyemura (1935) |  |
| *Anodonta imbecillis* (=*Utterbackia imbecillis* (Say, 1829)) **[U]** | Illinois, U.S.A. | Antipa and Small (1971) |  |
| *Anodonta implicata* (=*Utterbackiana implicata* (Say, 1829)) **[U]** | Woods Hole, Massachusetts; Lake Chautauqua, New York, U.S.A. | Kidder (1934) |  |
| *Anodonta suborbiculata* (=*Utterbackiana suborbiculata* (Say, 1831)) **[U]** | Illinois, U.S.A. | Antipa and Small (1971) |  |
| *Dreissena polymorpha* (Pallas, 1771) **[D]** | Slovakia | Zhang and Vďačný (2024) |  |
| *Anodontoides ferussacianus* (Lea, 1834) **[U]** | Illinois, U.S.A. | Antipa and Small (1971) |  |
| *Elliptio complanatus* (=*Elliptio complanata* ([Lightfoot], 1786)) **[U]** | North Carolina, U.S.A. | Beers (1962) |  |
| *Lasmigona complanate* (Barnes, 1823) **[U]** | Illinois, U.S.A. | Antipa and Small (1971) |  |
| *Lampsilis cariosa* (Say, 1817) **[U]** | Woods Hole, Massachusetts; Lake Chautauqua, New York, U.S.A. | Kidder (1934) |  |
| *Lampsilis radiata* (Gmelin, 1791) **[U]** | Woods Hole, Massachusetts; Lake Chautauqua, New York, U.S.A. | Kidder (1934) |  |
| *Lampsilis siliquoidea* (Barnes, 1823) **[U]** | Iowa; Illinois, U.S.A. | Penn (1958); Antipa and Small (1971) |  |
| *Lampsilis ventricosa* (=*Lampsilis cardium* Rafinesque, 1820) **[U]** | Illinois, U.S.A. | Antipa (1977); Antipa and Small (1971) |  |
| *Margaritifera falcata* (Gould, 1850) **[U]** | California, U.S.A. | Antipa et al. (2020) |  |
| *Pleurobema coccineum* (Conrad, 1836) **[U]** | Illinois, U.S.A. | Antipa and Small (1971) |  |
| *Sinanodonta woodiana* (Martens, 1877) **[U]** | | Slovakia | Zhang and Vďačný (2024) |
| *Tritogonia verrucosa* (Rafinesque, 1820) **[U]** | Illinois, U.S.A. | Antipa and Small (1971) |  |
| *Unio pictorum* (Linnaeus, 1758) and other *Unio* spp. **[U]** | Poland; Hungary; Bulgaria; Lake Ohrid, Yugoslavia | Raabe (1971) |  |
| *Uniomerus* sp. **[U]** | Illinois, U.S.A. | Antipa and Small (1971) |  |
| *Unio crassus* Philipsson, 1788 **[U]** | Slovakia | Zhang and Vďačný (2024) |  |
| **Hosts of *C. discophorus* Mermod, 1914** | |  |  |
| *Musculium lacustre* (Müller, 1774) **[S]** | Mazury, Poland | Dobrzańska (1958) |  |
| *Pisidium* sp. **[S]** | Jura Vaudois, Switzerland | Mermod (1914) |  |
| *Pisidium casertanum* (=*Euglesa casertana* (Poli, 1791)) **[S]** | Warszawa, Poland | Raabe (1971) |  |
| *Pisidium obtusale* (=*Euglesa obtusalis* (Lamarck, 1818)) **[S]** | Mazury, Poland | Dobrzańska (1958) |  |
| *Sphaerium corneum* (Linnaeus, 1758) **[S]** | Mazury, Poland | Dobrzańska (1958) |  |
| **Host of *C. acuminatus* (Clap. & Lachm., 1858), Raabe, 1933** | |  |  |
| *Dreissensia polymorpha* (=*Dreissena polymorpha* (Pallas, 1771)) **[D]** | Poland; Lake Balaton, Hungary; Lake Ohrid, Yugoslavia; Bulgaria; Copenhagen, Denmark, Slovakia | Raabe (1934, 1950, 1965); Fenchel (1965); Zhang and Vďačný (2024) |  |
| *Dreissensia bugensis* (Andrusov, 1897) **[D]** | Slovakia | Zhang and Vďačný (2024) |  |
| **Host of *C. klimentinus* Raabe, 1965** | |  |  |
| *Dreissensia polymorpha* (=*Dreissena polymorpha* (Pallas, 1771)) **[D]** | Lake Ohrid, Yugoslavia | Raabe (1965) |  |
| **Host of *C. magna* Kidder, 1934** | |  |  |
| *Elliptio complanatus* (=*Elliptio complanata* ([Lightfoot], 1786)) **[U]** | Woods Hole, Massachusetts, U.S.A. | Kidder (1934) |  |
| **Host of *C. paracurtus* sp. nov.** | |  |  |
| *Cristaria plicata* (Leach, 1814) **[U]** | | Lake Weishan, Shandong Province, China | present study |

^a^ Names of host organisms are listed as provided in the publications, followed by their current nomenclature in parentheses when applicable. The nomenclature of host organisms is according to MOLLUSCABASE (https://www.molluscabase.org/). The classification of *Conchophthirus* follows Raabe (1971). The higher classification of host organisms is provided in square brackets: D, Dreissenidae; S, Sphaeriidae; U, Unionida.

^b^ Only some representative literatures on valid *Conchophthirus* species are listed here and in total ten valid species were accepted by Raabe (1971).
